# Supplementary material for: Genome sequence diversity of SARS-CoV-2 obtained from clinical samples in Uzbekistan
Source: PLoS One. 2022 Jun 27;17(6):e0270314. doi: 10.1371/journal.pone.0270314 (PMC9236271; doi:10.1371/journal.pone.0270314)
Supplement: Attachment — Submitted filename: PONE-D-22-07071_IPnotes.pdf [file pone.0270314.s003.pdf]

PLOS ONE

Genome sequence diversity of SARS-CoV-2 obtained from clinical samples in Uzbekistan

--Manuscript Draft--

**Manuscript Number:**	PONE-D-22-07071
**Article Type:**	Research Article
**Full Title:**	Genome sequence diversity of SARS-CoV-2 obtained from clinical samples in Uzbekistan
**Short Title:**	Genome sequence diversity of SARS-CoV-2 in Uzbekistan
**Corresponding Author:**	Alisher A Abdullaev<br>Center for Advanced Technologies<br>Tashkent, Tashkent UZBEKISTAN
**Keywords:**	SARS-CoV-2, genome, delta variant of SARS-CoV-2
**Abstract:**	Tracking temporal and spatial genomic changes and evolution of the severe acute respiratory syndrome coronavirus 2 (SARS-CoV-2) are among the most urgent research topics worldwide, which help to elucidate the coronavirus disease 2019 (COVID-19) pathogenesis and the ~~level~~ of deleterious ~~effects~~. Our ~~current~~ study ~~concentrate~~s genetic diversity of SARS-CoV-2 variants in Uzbekistan and their associations with COVID-19 severity. Thirty-nine whole genome sequences (WGS) of SARS-CoV-2 isolated from PCR-positive patients from Tashkent, Uzbekistan for the period of July-August 2021, were generated and further subjected to ~~further~~ genomic analysis. Genome-wide annotations of clinical isolates from our study have revealed a total of 223 nucleotide-level variations including SNPs and 34 deletions at different positions throughout the entire genome of SARS-CoV-2. These changes included two novel mutations the Nonstructural protein (Nsp) 13: A85P and Nsp12: Y479N, which were unreported previously. There were two groups of co-occurred substitution patterns: the missense mutations in the Spike (S): D614G, Open Reading Frame (ORF) 1b: P314L, Nsp3: F924, 5`UTR:C241T; Nsp3:P2046L and Nsp3:P2287S, and the synonymous mutations in the Nsp4:D2907 (C8986T), Nsp6:T3646A and Nsp14:A1918V regions, respectively. The “Nextstrain” clustered the largest number of SARS-CoV-2 strains into the Delta clade (n=32; 82%), followed by two Alpha-originated (n=4; 10,3%) and 20A (n=3; 7,7%) clades. Geographically the Delta clade sample sequences were grouped into several clusters with the SARS-CoV genotypes from Russia, Denmark, USA, Egypt and Bangladesh. Phylogenetically, the Delta isolates in our study belong to the two main subclades 21A (56%) and 21J (44%). We found that females were more affected by 21A, whereas males by 21J variant ($\chi2=4.57$; $p \leq 0.05$, n=?). The amino acid substitution ORF7a:P45L in the Delta isolates found to be significantly associated with disease severity.
**Order of Authors:**	Alisher A Abdullaev<br>Abrorjon Abdurakhimov<br>Zebinisa Mirakbarova<br>Shakhnoza Ibragimova<br>Vladimir Tsoy<br>Sharofiddin Nuriddinov<br>Dilbar Dalimova<br>Shahlo Turdikulova, Professor<br>Ibrokhim Abdurakhmonov, Professor
**Additional Information:**	
**Question**	**Response**
**Financial Disclosure**	All authors of the manuscript (Alisher Abdullaev, Abror Abdurakhimov, Zebiniso

Powered by Editorial Manager® and ProduXion Manager® from Aries Systems Corporation

Enter a financial disclosure statement that describes the sources of funding for the work included in this submission. Review the submission guidelines for detailed requirements. View published research articles from *PLOS ONE* for specific examples.<br><br>This statement is required for submission and **will appear in the published article** if the submission is accepted. Please make sure it is accurate.<br><br>**Unfunded studies**<br>Enter: *The author(s) received no specific funding for this work.*<br><br>**Funded studies**<br>Enter a statement with the following details:<br>• Initials of the authors who received each award<br>• Grant numbers awarded to each author<br>• The full name of each funder<br>• URL of each funder website<br>• Did the sponsors or funders play any role in the study design, data collection and analysis, decision to publish, or preparation of the manuscript?<br>• **NO** - Include this sentence at the end of your statement: *The funders had no role in study design, data collection and analysis, decision to publish, or preparation of the manuscript.*<br>• **YES** - Specify the role(s) played.<br><br>* typeset	Mirakbarova, Shakhnoza Ibragimova, Vladimir Tsoy, Sharofiddin Nuriddinov, Dilbar Dalimova, Shakhlo Turdikulova Ibrokhim Abdurakhmonov) supported by the research grant from the Ministry of Innovative Development, Republic of Uzbekistan (Research Grant number: А-ИРВ-2021-125).<br><br>URL of Ministry of Innovative Development, Republic of Uzbekistan website: https://mininnovation.uz/en<br>The funders had no role in study design, data collection and analysis, decision to publish, or preparation of the manuscript.
**Competing Interests**<br><br>Use the instructions below to enter a competing interest statement for this submission. On behalf of all authors, disclose any competing interests that could be perceived to bias this work—acknowledging all financial support and any other relevant financial or non-financial competing interests.<br><br>This statement is required for submission and **will appear in the published article** if	The authors have declared that no competing interests exist.

<table>
<tr><td>the submission is accepted. Please make sure it is accurate and that any funding sources listed in your Funding Information later in the submission form are also declared in your Financial Disclosure statement.

View published research articles from PLOS ONE for specific examples.

NO authors have competing interests

Enter: The authors have declared that no competing interests exist.

Authors with competing interests

Enter competing interest details beginning with this statement:

I have read the journal's policy and the authors of this manuscript have the following competing interests: [insert competing interests here]

* typeset</td><td></td></tr>
<tr><td>Ethics Statement

Enter an ethics statement for this submission. This statement is required if the study involved:

• Human participants
• Human specimens or tissue
• Vertebrate animals or cephalopods
• Vertebrate embryos or tissues
• Field research

Write "N/A" if the submission does not require an ethics statement.

General guidance is provided below. Consult the submission guidelines for detailed instructions. Make sure that all information entered here is included in the Methods section of the manuscript.</td><td>Ethical approval for this study was obtained from Ethics Committee of Center for Advanced Technologies under Ministry of Innovative development (Approval Date: May 5, 2021; Approval Number CAT-EC-2021/05-1).</td></tr>
</table>

**Format for specific study types**<br><br>**Human Subject Research (involving human participants and/or tissue)**<br>• Give the name of the institutional review board or ethics committee that approved the study<br>• Include the approval number and/or a statement indicating approval of this research<br>• Indicate the form of consent obtained (written/oral) or the reason that consent was not obtained (e.g. the data were analyzed anonymously)<br><br>**Animal Research (involving vertebrate animals, embryos or tissues)**<br>• Provide the name of the Institutional Animal Care and Use Committee (IACUC) or other relevant ethics board that reviewed the study protocol, and indicate whether they approved this research or granted a formal waiver of ethical approval<br>• Include an approval number if one was obtained<br>• If the study involved *non-human primates*, add *additional details* about animal welfare and steps taken to ameliorate suffering<br>• If anesthesia, euthanasia, or any kind of animal sacrifice is part of the study, include briefly which substances and/or methods were applied<br><br>**Field Research**<br><br>Include the following details if this study involves the collection of plant, animal, or other materials from a natural setting:<br>• Field permit number<br>• Name of the institution or relevant body that granted permission	
**Data Availability**<br><br>Authors are required to make all data underlying the findings described fully available, without restriction, and from the time of publication. PLOS allows rare exceptions to address legal and ethical concerns. See the PLOS Data Policy and FAQ for detailed information.	Yes - all data are fully available without restriction

A Data Availability Statement describing where the data can be found is required at submission. Your answers to this question constitute the Data Availability Statement and **will be published in the article**, if accepted.<br><br>**Important:** Stating 'data available on request from the author' is not sufficient. If your data are only available upon request, select 'No' for the first question and explain your exceptional situation in the text box.<br><br>Do the authors confirm that all data underlying the findings described in their manuscript are fully available without restriction?	
**Describe where the data may be found in full sentences. If you are copying our sample text, replace any instances of XXX with the appropriate details.**<br><br>• If the data are **held or will be held in a public repository**, include URLs, accession numbers or DOIs. If this information will only be available after acceptance, indicate this by ticking the box below. For example: *All XXX files are available from the XXX database (accession number(s) XXX, XXX.*).<br>• If the data are all contained **within the manuscript and/or Supporting Information files**, enter the following: *All relevant data are within the manuscript and its Supporting Information files.*<br>• If neither of these applies but you are able to provide **details of access elsewhere**, with or without limitations, please do so. For example:<br><br>*Data cannot be shared publicly because of [XXX]. Data are available from the XXX Institutional Data Access / Ethics Committee (contact via XXX) for researchers who meet the criteria for access to confidential data.*<br><br>*The data underlying the results presented in the study are available from (include the name of the third party*	The data underlying the results presented in the study are available from FlagShare Public Repository https://doi.org/10.6084/m9.figshare.19221276.v1

and contact information or URL).<br>• This text is appropriate if the data are owned by a third party and authors do not have permission to share the data.<br><br>* typeset	
Additional data availability information:	Tick here if the URLs/accession numbers/DOIs will be available only after acceptance of the manuscript for publication so that we can ensure their inclusion before publication.

Manuscript Click here to access/download;Manuscript;Manuscript-SARS-CoV2-rev.docx

Title: Genome sequence diversity of SARS-CoV-2 obtained from clinical samples in Uzbekistan

Authors: Alisher Abdullaev[1*], Zebiniso Mirakbarova[1], Shakhnoza Ibragimova[1], Vladimir Tsoy[1], Sharofiddin Nuriddinov[1], Dilbar Dalimova[1], Shakhlo Turdikulova[1], Ibrokhim Abdurakhmonov[1,2]

Affiliations: [1]Center for Advanced Technologies, Talabalar shaharchasi 3a, 100174 Tashkent, Uzbekistan; [2]Center of Genomics and Bioinformatics, Academy of Sciences of Uzbekistan, Qibray region, University street - 2, Tashkent 111215, Republic of Uzbekistan.

*Corresponding author

Email: abdullaev_alisher@yahoo.com

Abstract

Tracking temporal and spatial genomic changes and evolution of the severe acute respiratory syndrome coronavirus 2 (SARS-CoV-2) are among the most urgent research topics worldwide, which help to elucidate the coronavirus disease 2019 (COVID-19) pathogenesis and the level of deleterious effects. Our current study concentrates genetic diversity of SARS-CoV-2 variants in Uzbekistan and their associations with COVID-19 severity. Thirty-nine whole genome sequences (WGS) of SARS-CoV-2 isolated from PCR-positive patients from Tashkent, Uzbekistan for the period of July-August 2021, were generated and further subjected to further genomic analysis. Genome-wide annotations of clinical isolates from our study have revealed a total of 223 nucleotide-level variations including SNPs and 34 deletions at different positions throughout the entire genome of SARS-CoV-2. These changes included two novel mutations the Nonstructural protein (Nsp) 13: A85P and Nsp12: Y479N, which were unreported previously. There were two groups of co-occurred substitution patterns: the missense mutations in the Spike (S): D614G, Open Reading Frame (ORF) 1b: P314L, Nsp3: F924, 5`UTR:C241T; Nsp3:P2046L and Nsp3:P2287S, and the synonymous mutations in the Nsp4:D2907 (C8986T), Nsp6:T3646A and Nsp14:A1918V regions, respectively. The "Nextstrain" clustered the largest number of SARS-CoV-2 strains into the Delta clade (n=32; 82%), followed by two Alpha-originated (n=4; 10,3%) and 20A (n=3; 7,7%) clades. Geographically the Delta clade sample sequences were grouped into several clusters with the SARS-CoV genotypes from Russia, Denmark, USA, Egypt and Bangladesh. Phylogenetically, the Delta isolates in our study belong to the two main subclades 21A (56%) and 21J (44%). We found that females were more affected by 21A, whereas males by 21J variant ($\chi 2=4.57$; $p \leq 0.05$, n=?). The amino acid substitution ORF7a:P45L in the Delta isolates found to be significantly associated with disease severity.

Keywords: SARS-CoV-2, genome, delta variant of SARS-CoV-2

Author Summary

The virus SARS-CoV-2 that causes COVID-19 is subject to mutations and is constantly changing, because of which new variants of the virus regularly emerging. In some countries, the SARS-CoV-2 changes rapidly leading to the novel variants of concern (VOC) such as Delta or Omicron. The objective of current work was to study the genetic diversity of a novel coronavirus SARS-CoV-2 distributed in Uzbekistan using a whole genome sequencing (WGS). We successfully identified main genotypes and the mutation profiles of the SARS-CoV-2 variants distributed in the country during the second infection waive with a possible impact on the infectivity and disease severity of COVID-19. We found that some of Delta variants affect more women than men. Our results revealed two novel mutations and one amino acid substitution leading to a weakening of the virus in terms of disease severity. These findings should be potentially useful for the scientific community in terms of disease diagnostics, monitoring and treatment.

Highlights

- ✓ Genome-wide annotations and diversity of SARS-CoV-2 clinical isolates from Uzbekistan are reported for the first time
- ✓ Phylogenetic analysis revealed several variants with 82% prevalence of Delta variant
- ✓ Delta isolates from Uzbekistan belong to the two main subclades 21A and 21J
- ✓ Two groups of co-occurred genomic substitution patterns are identified
- ✓ Novel mutations such as Nsp13: A85P and Nsp12: Y479N are identified

- ✓ ORF7a: P45L substitution has been found to be significantly associated with disease severity.

Introduction

A novel coronavirus, SARS-CoV-2, was first identified in December 2019 (Wu et al., 2020) and rapidly spread around the world. According to Johns Hopkins Coronavirus Resource Center (CRC) SARS-CoV-2 infected hundreds of millions and killed more than 5 million of people until the middle of 2021 [1]. Numerous whole genome sequence (WGS) analysis found mutational variations in the viral genome [2-5].

Currently, there a need for increased global sequencing efforts to identify the spread of new variants as quickly as possible since analyzing genome sequencing and single-nucleotide polymorphism (SNP) calling have been a hotspot for a wide variety of epidemiological, clinical, and therapeutic [2, 3, 7, 8]. Although most mutations in the SARS-CoV-2 genome are expected to be either deleterious or relatively neutral, a small proportion will affect functional properties and may alter infectivity, disease severity in even after the host immunity background [2, 9, 10].

Genomic variation ~~has also been shown to be~~ informative ~~in terms of~~ tracking the distribution of the virus [11] and identifying major clades related to the various variants of SARS-CoV-2 with different epidemiological outcome [12]. The genomic variability of SARS-CoV-2 specimens scattered across the globe can underly geographically specific etiological effects. Previous studies ~~have~~ revealed the prevalence of single nucleotide transitions as the major mutational type across the world characterized by geographic and genomic specificity [2].

Country of origin and time since the start of the pandemics were the most influential metadata associated with genomic variation. It was highlighted that some geographic

regions (populations) have unusually high (many new variants) while others have low (isolated) viral phylogenetic diversity. Such studies provided a direction to prioritize genes associated with outcome predictors (e.g., health, therapeutic, and vaccine outcomes) and to improve DNA tests for predicting disease status [13]. Current study is aimed at identifying SARS-CoV-2 variants and mutation profile in the coronavirus genome presented during the second wave of coronavirus pandemic in Uzbekistan and assessing their impact on the disease severity.

Overall pattern of the CVOID-19 pandemic in Uzbekistan, since the first confirmed case reported on 15th of March, 2020, has accompanied two distinct disease waves until October of 2021. Until the 20th of October, 2021, a total of 182 060 cases and 1292 deaths were reported, of that the first peak occurred in July-August 2020, when within the two months, the total number of cases increased rapidly from 12 295 to 43 476 with two months increase of 31 181. The second waive has occurred in July to August of 2021, following the increases of infected people from 113 559 to 160 589 within the two (n=47 030) months period (Fig.1; https://www.worldometers.info). During these two diseases waives, a number of deaths was 299 and 367, respectively, and was accounted for 30.5% of total deaths (n=2179) since pandemics started in Uzbekistan (Fig.2; https://www.worldometers.info). The lowest number of detected CVOID-19 cases in Uzbekistan (n=1078) was observed in February 2021 (WHO, https://covid19.who.int/region/euro/country/uz).

Fig.1. COVID-19 cases in Uzbekistan since March 2020 to September 2021 (according to Worldometer, https://www.worldometers.info).

Fig.2. Deaths caused by COVID-19 in Uzbekistan since March 2020 to September 2021 (according to Worldometer, https://www.worldometers.info).

Previous efforts [14], have characterized 18 high-quality WGS reads for SARS-COV-2 from the very early symptomatic COVID-19 patients, sampled from Tashkent region clinics for the period of October and beginning of December, 2020. After the first wave, WGS of 18 SARS-CoV-2 genomes, distributed in Uzbekistan at that period, have revealed a total of 128 SNPs, consisting of 45 shared and 83 unique mutations, phylogenetically suggesting their origin and spread from European and Near East countries because of international travel~~s~~ [14]. However, a periodic genome sequencing of SARS-CoV-2 should be carried out to identify the degree of mutations and novel variants of concern (VOC), potentially useful for disease diagnostics, monitoring and treatment.

Therefore, the main purpose of current study was to initiate another large-sample, WGS and genetic diversity evaluation study of the SARS-CoV-2, distributed during the second infection waive period in Uzbekistan, using virus samples isolated from CVOID-19 positive patients with mild, moderate, and severe symptoms. Here we report that in the second COVID-19 disease-waive, a SARS-CoV-2 has changed into several new variants with majority (82%) distribution of Delta variant in the country. We have identified 223 nucleotide-level variations and 34 deletions at different positions with the two co-occurred genomic substitution patterns and two novel mutations (Nsp13: A85P and Nsp12: Y479N). Results showed that one of missense substitutions namely ORF7a: P45L ~~has been found to be~~ significantly associated with disease severity.

Results

Genome-wide characterization of SARS-CoV-2 isolates from Uzbekistan

Forty-eight samples collected from the COVID-19 positive patients after qPCR screening were selected for further WGS. Out of 48 samples isolated from SARS-CoV-2 positive patients, we generated 39 high-quality sequences. The average genome read length of 39 samples was 29 886.5 (σ=4.3) nucleotides, the average content of nucleotide was 29.93% of Adenine (A), 32.11% of Thymidine (T), 19.60% of Guanine (G) and 18.32% of Cytosine (C). The GC content was 37.92 percent.

Genome-wide annotations of SARS-Cov-2 positive sample isolates in our study revealed a total of 179 nucleotide-level variations including two novel mutations, which were unreported previously, and 34 deletions at different positions throughout the entire genome of SARS-CoV-2. Comparative sequence alignment of 179 mutations to the reference genome determined that 114 (63.7%) were missense (nonsynonymous) mutations and 65 (36.3%) were silent (synonymous) mutations. The average number of SNVs per isolate was 31.6 (σ =9.23) with minimum and maximum values of 9 and 45 respectively (Table 1; Fig. 3).

Table 1. The average number of nucleotide and amino acid substitutions, and deletions per the SARS-CoV-2 genome among 39 samples identified in Uzbekistan (with respect to the reference genome NC_045512.2).

Values	nucleotide substitutions	nucleotide deletions	amino acid substitutions	amino acid deletions
Average	31.61	16.46	25.46	5.15
SD	9.23	4.38	7.35	1.46
max	45	25	34	8

min	9	13	7	4

Fig.3. Box plot of nucleotide and amino acid substitutions, and deletions per the SARS-CoV-2 genome among 39 samples identified in Uzbekistan (with respect to the reference genome NC_045512.2). The cross marks the mean values, the bold line marks median

Analysis of nucleotide substitutions rate in the SARS-CoV-2 genome showed prevalence of transitions of 68.1% with the most common C>T substitution of 42% (Fig. 4 and 5; Table 2). The average number of transitions per isolate was 21. 1 (σ=6.68), with a minimum and maximum number of 6 and 33, respectively (Fig.5). Among transversion events a G>T substitutions had the highest rate of 16% (Table 2; Fig.4).

Table 2. Single nucleotide substitutions frequency in the genome of SARS-CoV-2 isolates (n=39) from Uzbekistan.

Nucleotide substitution	Type	Average per genome	Frequency
C>T	Transition	13.10	0.42
G>T	Transversion	4.90	0.16
A>G	transition	3.23	0.10
T>C	transition	2.49	0.08
G>A	transition	2.33	0.08
C>G	transversion	1.69	0.05
C>A	transversion	1.18	0.04
T>G	transversion	0.97	0.03
G>C	transversion	0.38	0.01

A>T	transversion	0.31	0.01
A>C	transversion	0.26	0.01
T>A	transversion	0.21	0.01

Fig. 4. Box plot of distribution of single nucleotide substitutions per genome among the SARS-CoV-2 isolates (n=39) in Uzbekistan. The cross marks the mean values, the bold line marks median

Fig. 5. Box plot of distribution of transitions and transversions per genome among the SARS-CoV-2 isolates (n=39) in Uzbekistan. The cross marks the mean values, the bold line marks median

Detailed analysis on genomic distribution of SNPs and amino acid substitutions revealed that the most variable regions are the ORF1a and ORF1b with 59 and 43 SNPs, respectively (Table 3). Interestingly, the silent SNPs were more abundant in the Nsp3 and Nsp12 (RdRp), which were 8 and 10, respectively. The highest number of amino acid substitutions was found in the ORF1a (n=34), ORF1b (n=24) and S (24) genes (Table 3). The only one amino acid substitution I82T was present in the Membrane (M) protein (n=29; 74% of isolates) and no mutation found in the Envelope (E) protein. Among all genes with the highest rate of amino acid substitutions was the Spike (S) gene (6 mutations with the frequency of occurrence from 79% to 100%) and the ORF1b (3 mutations with the frequency of occurrence from 77% to 97%). In the S protein, the SARS-CoV-2 mutation D614G was identified in all isolates (n=39; 100%) from our study, followed by R158G (n=35; 90%), T19R (n=34; 87%), D950N (n=33; 85%), L452R (n=33;

85%), T478K (n=32; 82%) and P681R (n=31; 79%). Frequencies of all identified missense mutation in SARS-CoV-2 isolates from our study have been presented in the Table 4.

Table 3. Genome distribution of the total number of identified nucleotide and amino acid substitutions among the SARS-CoV-2 (n=39) samples from Uzbekistan.

Gene/region	Total number of SNVs (silent)	Total number of amino acid substitutions
5`UTR	3	-
3`UTR	2	-
ORF1a	59 (25)	34
ORF1b	43 (19)	24
ORF3a	5 (0)	5
ORF7a	6 (1)	5
ORF7b	2 (0)	2
ORF8	6 (2)	4
ORF9b	4 (1)	3
M	3 (2)	1
N	12 (1)	11
S	32 (8)	24

Table 4. The frequency of missense mutation in the SARS-CoV-2 genotypes identified in Uzbekistan (nt and aa positions are indicated relative to the reference genome NC_045512.2).

Gene	Gene product/region	SNP (refseq position)	AA substitution	Number of samples	Frequency
	Nsp2	C884T	R207C	22	0.56
	Nsp3	C6402T	P2046L	16	0.41
	Nsp3	C7124T	P2287S	16	0.41
	Nsp4	C10029T	T3255I	16	0.41
	Nsp6	A11201G	T3646A	15	0.38
	Nsp3	G4181T	A1306S	14	0.36
	Nsp4	G9053T	V2930L	14	0.36
	Nsp4	C9891T	A3209V	11	0.28
	Nsp3	C5184T	P1640L	9	0.23
	Nsp6	T11418C	V3718A	9	0.23
	Nsp3	C6539T	H2092Y	8	0.21
	Nsp6	C11195T	L3644F	7	0.18
	Nsp3	C3096T	S944L	7	0.18
	Nsp2	G1048T	K261N (K81N)	6	0.15
	Nsp2	C1191T	P309L	6	0.15
	Nsp3	T6954C	I2230T	4	0.10
	Nsp3	C3267T	T1001I	3	0.08
ORF1a	Nsp3	C5388A	A1708D	2	0.05

Nsp3	C5031T	T1589I	2	0.05
Nsp3	C4455T	A1397V	1	0.03
Nsp6	T11073C	F3603S	1	0.03
Nsp2	G1820A	G519S	1	0.03
Nsp10	T13188C	I4308T	1	0.03
3C-like proteinase	A10323G	K3353R	1	0.03
Nsp3	C4613T	L1450F	1	0.03
Nsp2	T1227G	M321R	1	0.03
Nsp8	T12667G	N4134K	1	0.03
Nsp2	G1161A	R299K	1	0.03
Nsp3	C5826T	T1854I	1	0.03
Nsp9	C12756T	T4164I	1	0.03
Nsp3	G6446T	V2061F	1	0.03
Nsp6	G11417A	V3718I	1	0.03
Nsp8	G12569T	V4102L	1	0.03
Nsp2	T3026A	Y921N	1	0.03

	C14408T	P314L	39	1
	C16466T	P1000L	33	0.85
	G15451A	G662S	30	0.77

		C19220T	A1918V	18	0.46
		C20320T	H2285Y	6	0.15
		T19420C	S1985P	4	0.10
	ORF1ab	C16349T	S961L	4	0.10
ORF1b	polyprotein	A21137G	K2557R	3	0.08
		A17431G	I1322V	2	0.05
		C18176T	P1570L	2	0.05
		G16489C†	A1008P† (NSP13: A85P)	1	0.03
		G18040T	A1525S	1	0.03
		G18469A	D1668N	1	0.03
		G19788C	E2107D	1	0.03
		T16307C	F947S	1	0.03
		G16852T	G1129C	1	0.03
		G21204T	K2579N	1	0.03
		C17125T	L1220F	1	0.03
		C14120T	P218L	1	0.03
		A20059G	S2198G	1	0.03
		G16741T	V1092F	1	0.03
		G20578T	V2371L	1	0.03

		G13726A	V87I	1	0.03
		T14875A†	Y470N† (NSP12: Y479N)	1	0.03
		C25469T	S26L	31	0.79
		A25439C	K16T	3	0.08
3a	ORF3a protein	G25947C	Q185H	2	0.05
		T25520G	F43C	1	0.03
		G25996T	V202L	1	0.03
		C27752T	ORF7a:T120I	31	0.79
		T27638C	ORF7a:V82A	23	0.59
7a	ORF7a protein	C27739T	ORF7a:L116F	11	0.28
		C27527T	ORF7a:P45L	7	0.18
		G27478T	ORF7a:V29L	1	0.03
7b	ORF7b protein	C27874T	ORF7b:T40I	14	0.35
		T27835C	ORF7b:I27T	1	0.03
		A28095T	ORF8:K68*	4	0.10

8	ORF8 protein	A28111G	ORF8:Y73C	4	0.10
		C27972T	ORF8:Q27*	3	0.08
		G28048T	ORF8:R52I	1	0.03
		A28461G	ORF9b:T60A	33	
9b	ORF9b protein	C28291T	ORF9b:P3L	4	0.10
		G28396A	ORF9b:G38D	1	0.03
M	Membrane glycoprotein	T26767C	I82T	29	0.74
	Nucleocapsid phosphoprotein				
	NTD: RBD	A28461G	D63G	33	0.85
	C-tail	G29402T	D377Y	32	0.82
	SR-R	G28881T	R203M	30	0.77
N	LQ-R	G28916T	G215C	15	0.38
	C-tail	G29427A	R385K	9	0.23
		G28280C			
	N-tail	A28281T			
		T28282A	D3L	4	0.10

LQ-R	C28977T	S235F	4	0.10
CTD	C29358T	T362I	4	0.10
SR-R	G28883C	G204R	3	0.08
SR-R	G28881A, G28882A	R203K	3	0.08
NTD	G28739T	A156S	1	0.03
LQ-R	G28975A	M234I	1	0.03
Spike protein				
S1: SD2	A23403G	D614G	39	1.00
S1: NTD	del22029-22034	R158G	35	0.90
S1: NTD	C21618G	T19R	34	0.87
S2: HR1	G24410A	D950N	33	0.85
S1: RBD: RBM	T22917G	L452R	33	0.85
S1: RBD: RBM	C22995A	T478K	32	0.82
S1: SD2	C23604G	P681R	31	0.79
S1: RBD: RBM	A23063T	N501Y	7	0.18
S1: NTD	C21846T	T95I	7	0.18
S1: NTD	G21987A	G142D	5	0.13
S1: SD1	C23271A	A570D	4	0.10

S	S2	G24914C	D1118H	4	0.10
	S2	A24110C	I850L	4	0.10
	S1: SD2	C23604A	P681H	3	0.08
	S2: HR1	T24506G	S982A	3	0.08
	S2	C23709T	T716I	3	0.08
	S1	A22320C	D253A	1	0.03
	S2: TM	G25229A	G1223S	1	0.03
	S2	T24903C	I1114T	1	0.03
	S1: RBD	A22814G	I418V	1	0.03
	S2	G24751T	L1063F	1	0.03
	S1: NTD	C22323T	S254F	1	0.03
	S1: SD2	T23600C	S680P	1	0.03
	S2: TM	G25250T	V1230L	1	0.03

Nsp – nonstructural protein; ORF – open reading frame; S1, S2 - Spike subunits 1 and 2; NTD - N-terminal domain; RBD - Receptor Binding Domain; RBM – Receptor Binding Motif; SD1, SD2 - the subdomains 1 and 2; HR1- heptad repeats; TM - transmembrane domain; SR-R – Serine/Arginine-rich region; LQ-R - Leucin/Glutamine-rich region; CTD - C-terminal domain; * – nonsense mutation; †-novel, unreported substitutions

The other high frequency mutations (identified in 100% of samples) were the P314L amino acid change mutation in the ORF1b, the synonymous F924 (C3037T) change in the Nsp3 and the nucleotide substitution C241T in the 5`UTR (Table 4). Thus,

in all isolates, regardless of their phylogenetic position, the substitution of D614G co-occurred within the ORF1b: P314L, Nsp3: F924 and 5`UTR: C241T. Further co-occurrence analysis revealed of the following 5 mutations: Nsp3: P2046L, Nsp3: P2287S, synonymous in the Nsp4: D2907 (C8986T), Nsp6: T3646A and Nsp14: A1918V.

Analysis of mutation profile among the symptomatic isolates from our study have revealed the two novel nucleotide substitutions G16489C and T14875A, which resulted in amino acid changes in the Nsp13: A85P (ORF1b: A1008P) and Nsp12: Y479N (ORF1b: Y470N), respectively (Table 4; GISAID IDs: EPI_ISL_3188967, EPI_ISL_3188979).

Genome-wide characterization of SARS-CoV-2 isolates has identified 34 nucleotide deletions resulted in 11 amino acid deletions (at the Spike, ORF8, Nsp6 and Nsp1 regions) and non-coding deletion g.a28271 at the upstream of the N gene (Table 5). This non-coding deletion g.a28271 has been identified in all SARS-CoV-2 isolates (n=39; 100%). The average number of nucleotide deletions was 16.5 (σ =4.38) per isolate with the minimum and maximum of 13 and 25, respectively. The average number of amino acid deletions was 5.15 (σ =1.46) per isolate with the minimum and maximum of 4 and 8, respectively (Tables 1 and 5; Fig.3).

Tab.5. The frequency of deletions identified among the SARS-CoV-2 isolates (n=39) in Uzbekistan.

nt deletion	Genomic region	aa deletion	Samples	Freq
g.a28271-	noncoding, upstream N gene	-	39	1.00
22029-22034	S	EF156-157Δ	35	0.90
28248-28253	ORF8	DF119-120Δ	33	0.85
21992-21994	S	Y144Δ	19	0.49

11288-11296	NSP6	SGF 3675-3677Δ	11	0.28
21765-21770	S	HV69-70Δ	5	0.13
516-518	NSP1	M85Δ	1	0.03

Positions are indicated relative to the reference genome (NC_045512.2)

The frequency of amino acid deletions in non-structural proteins were 85% in the ORF8: D119/F120Δ (n=33), 28% in the Nsp6: SGF3675-3677Δ (n=11) and 3% in the Nsp1: M85Δ (n=1). In the S protein there were three known amino acid deletions with the following occurrence frequencies:90% in for the E156/F157Δ (n=35; 90%), 49% for the Y144 Δ (n=19) and 13% for the HV69-70Δ (n=5 Table 5).

Phylogeny of SARS-CoV-2 isolates from Uzbekistan

The Nextclade-based phylogenetic analysis of the SARS-CoV-2 genome samples sequenced in our study has revealed that the most currently distributed SARS-CoV-2 variants in our dataset belong to the tree clades: 20A (Alpha, V1), 20I (Alpha, V1), and 21A (Delta) in consonance with "Nextstrain"'s nomenclature system (Hadfield et al, 2018). Neighbor Joining analysis and the "Nextstrain" classification has grouped 82% of our SARS-CoV-2 sample genome sequences into the Delta variant clade (n=32; 82%), followed by Alpha (n=4; 10.3%) and 20A (n=3; 7.7%) clades (Figs. 6 and 7).

Fig.6. The Nextclade based phylogenetic tree of the SARS-CoV-2 variants isolated in Uzbekistan. Sequences are placed on a reference tree, clades were assigned to the nearest neighbor, and private mutations analyzed. Brunches with colored circle represents variants from Uzbekistan.

Fig. 7. Neighbor-joining phylogenetic tree of the SARS-CoV-2 isolates from Uzbekistan. Samples are colored by taxonomic affiliation to clades or subclades. Clades

are assigned according to Nextclade nomenclature. Delta variant divided to subclades 21A (green) and 21J (orange). Blue color - clade 20A, Red color – clade 20I (Alpha variant). Bootstrap values were shown.

The Nexstclade phylogenetic analysis based on the WGS showed that Alpha clade variants from our dataset, namely EPI_ISL_3188994 and EPI_ISL_3188999, have clustered with the SARS-COv-2 genome sequences from France and Armenia, whereas EPI_ISL_3188965 and EPI_ISL_3188992 from our study have clustered with England coronavirus sample sequences. The clade 20A sequences from our dataset such as EPI_ISL_3188963, EPI_ISL_3188985 and EPI_ISL_3188990 clustered with the SARS-CoV-2 variants sequenced from USA and England. Our Delta clade grouped sequence reads were clustered with coronavirus sample sequence reads from Russia, Denmark, USA, Egypt and Bangladesh.

Phylogeny and genomic characterization of the Delta isolates from Uzbekistan

Since most of the isolates were classified to the Delta clade, further step was to differentiate them by variations in the S protein sequence and other genomic regions. Analysis of the S gene mutation profile among Delta variants from our study has revealed a total 31 mutations, including five amino acid deletions and eight synonymous SNP mutations. In the Spike protein, high frequency mutations, covering the 94-100% of samples sequenced, were T19R, EFR156-158G, L452R, T478K, D614G, P681R and D950N (Table 6). Along with above variants, there were several genotypes that also have contained Y144Δ (38%), T95I (22%), G142D (16%) and I850L (13%) variations in the Spike protein (Table 6). The Delta genotypes sampled from Uzbekistan in our study were

also characterized by occurrence of the high frequency (91%-100%) of nonsynonymous changes in other genomic regions, namely at the ORF1b: P314L, G662S and P1000L, ORF3a: S26L, ORF7a: T120I ORF9b: T60A, N: D63G, N: D377Y, N: R203M, and M: I82T (Table 7).

Table 6. Frequency of the identified Spike protein mutations (missense/synonymous) and deletions in the Delta isolates from Uzbekistan.

aa position	Reference	Variant	nt position	mutation	total	Freq	Type
19	T	R	21618	T>G	32	1.00	missense
69	H	ΔH	21765-21768	Δ	1	0.03	deletion
70	V	ΔV	21769-21770	Δ	1	0.03	deletion
95	T	I	21618	C>G	7	0.22	missense
142	G	D	21987	G>A	5	0.16	missense
144	Y	ΔY	21992-21994	Δ	12	0.38	deletion
156	E	ΔE	22029-22030	Δ	32	1.00	deletion
157	F	ΔF	22031-22033	Δ	32	1.00	deletion
158	R	G	22034	Δ	32	1.00	missense
253	D	A	22320	A>C	1	0.03	missense

297	S	S	22453	A>C	1	0.03	synonymous
302	T	T	22468	G>T	1	0.03	synonymous
312	I	I	22498	C>T	1	0.03	synonymous
375	S	S	22687	C>A	2	0.06	synonymous
418	I	V	22814	A>G	1	0.03	missense
452	L	R	22917	T>G	30	0.94	missense
478	T	K	22995	C>A	31	0.97	missense
501	N	Y	23063	A>T	1	0.03	missense
614	D	G	23403	A>G	32	1.00	missense
665	P	P	23557	A>T	1	0.03	synonymous
680	S	P	23600	T>C	1	0.03	missense
681	P	R	23604	C>G	31	0.97	missense
808	D	D	23986	T>C	1	0.03	synonymous
850	I	L	24110	A>C	4	0.13	missense
950	D	N	24410	G>A	32	1.00	missense
982	S	A	24506	T>G	1	0.03	missense
1061	V	V	24745	C>T	8	0.25	synonymous
1063	L	F	24751	G>T	1	0.03	missense

1223	G	S	25229	G>A	1	0.03	missense
1230	V	L	25250	G>T	1	0.03	missense
1238	T	L	25276	C>T	1	0.03	synonymous

Positions are indicated relative to the reference genome (NC_045512.2)

Table 7. Mutations in the Delta isolates (21A and 21J) with the frequency of occurrence higher than 20%.

nt position	variant	total	frequency
A23403G	S: D614G	32	1.00
C14408T	ORF1b: P314L	32	1.00
del22029-22034	S: R158G	32	1.00
C21618G	S: T19R	32	1.00
A28461G	N: D63G	32	1.00
A28461G	ORF9b: T60A	32	1.00
G24410A	S: D950N	32	1.00
G29402T	N: D377Y	32	1.00
C16466T	ORF1b: P1000L	31	0.97
C22995A	S: T478K	31	0.97
C23604G	S: P681R	31	0.97
C25469T	ORF3a: S26L	31	0.97
C27752T	ORF7a: T120I	31	0.97
T22917G	S: L452R	30	0.94
G28881T	N: R203M	29	0.91

G15451A	ORF1b: G662S	29	0.91
T26767C	M: I82T	29	0.91
T27638C	ORF7a: V82A	23	0.72
C884T	ORF1a: R207C	18	0.56
C19220T	ORF1b: A1918V	18	0.56
C6402T	ORF1a: P2046L	16	0.50
C7124T	ORF1a: P2287S	16	0.50
C10029T	ORF1a: T3255I	16	0.50
G28916T	N: G215C	16	0.50
A11201G	ORF1a: T3646A	16	0.50
G4181T	ORF1a: A1306S	14	0.44
G9053T	ORF1a: V2930L	14	0.44
C27874T	ORF7b: T40I	14	0.44
C9891T	ORF1a: A3209V	11	0.34
C27739T	ORF7a: L116F	11	0.34
G29427A	N: R385K	9	0.28
C5184T	ORF1a: P1640L	9	0.28
T11418C	ORF1a: V3718A	9	0.28
C6539T	ORF1a: H2092Y	8	0.25
C11195T	ORF1a: L3644F	7	0.22
C3096T	ORF1a: S944L	7	0.22
C27527T	ORF7a: P45L	7	0.22
C21846T	S: T95I	7	0.22

According to the Nexststrain phylogenetic grouping based on the amino acid and several nucleotide substitutions, the Delta isolates from our study were divided into the two main subclades 21A (n=18; 56.2%) and 21J (n=14; 43.8 (Figs. 6-7). All isolates in the subclade 21J were mainly defined by a frequently observed synonymous nucleotide substitution C8986T, and the amino acid substitutions at the ORF1a: A1306S (G4181T), P2046L (C6402T), P2287S (C7124T), V2930L (G9053T), T3255I (C10029T), T3646A (A11201G), ORF1b: A1918V (C19220T), and ORF7b: T40I (C27874T). Three of them (ORF1a: A1306S, V2930L, and ORF1b: A1918V) were unique to the 21J subclade, while the rest were present of subclade 21A, formed by two (11%) of sample genomes sequenced. In the subclade 21J, there were also 50% of isolates bearing the S protein - specific mutation T95I (C27874T).

Isolates in the subclade 21J were characterized by a higher number of average nucleotide and amino acids substitutions, but lower nucleotide and amino acids deletions per isolate compared to the subclade 21A (Table 8, Fig. 8). The broader variation range of mutations events was observed among isolates in the subclade 21A (Table 8, Fig. 8) than the subclade 21J. It was found that the substitutions N: R385K, ORF1a: S944L, H2092Y, L3644F, and ORF1b: H2285Y were always co-occurred and present in 50% of isolates in the subclade 21A of our study. Analysis of geographical distribution indicated that all samples in the subclade 21A were mostly clustered close to the SARS-CoV-2 genotypes from Bangladesh, whereas all samples in the subclade 21J were mostly clustered with the genome sequence samples of coronavirus from Egypt and Russia.

Fig. 8. Distribution of mutation types in the Delta variant subclades 21A (n=18) and 21J (n=14).

Table 8. Sample mean values of nucleotide and amino acid mutation type between the subclades 21A and 21J.

Types	21J (n=14)	σ^2	21A (n=18)	σ^2
nt substitutions	36.57 (SD=2.31)	4.96	30.56 (SD=8.94)	75.58
nt deletions	14.29 (SD=32)	9.92	17.00 (SD=4.93)	23.48
aa substitutions	31.36 (SD=1.86)	3.23	24.11 (SD=5.69)	30.65
aa deletions	4.43(SD=1.08)	1.10	5.30 (SD=1.64)	2.55

Analysis of disease severity among patients infected with the Delta variant

The gender distribution of selected patients in our study was 51.3% (n=20) of males, and 48.7% (n=19) of females, with age distribution of 50 (SD=14.7) and 60.7 (SD=13.2) years respectively, the mean age of all 39 patients in both gender groups was 55.2 (SD=14.9) (Table 10; Fig. 9).

Fig. 9. Age distribution among COVID-19 patients

A number of patients with mild, severe, and critical manifestation of COVID-19 were 46.2% (n=18), 33.3% (n=13), and 20.5% (n=8) respectively. No statistically significant associations were found between disease severity vs. clades/subclades (p=0.32), and between disease severity vs. gender (p=0.4).

Clinical symptoms among patients affected by the Delta variant (n=32) were distributed by the disease severity to three groups: mild 46.9% (n=15), severe 31.2% (n=10), critical 21.9% (n=7), and death (2.5% of all studied). Almost all hospitalized patients in three groups had at least two chronic comorbidities, including obesity, cardiovascular diseases, and diabetes. Examining the effect of comorbidities on a clinical outcome, there were no significant associations found among the above-mentioned three groups related to comorbidities and disease severity. Interestingly, a distribution analysis of the Delta subclades among gender groups revealed that females were more affected by the 21A, whereas males by the 21J variants (OR=4.73; 95% CI 1.05 - 24.44; $\chi 2=4.57$; p=0.032; Fig. 10). There were no significant differences found between gender and disease severity (p=0.4).

Fig. 10. Distribution of SARS-CoV-2 Delta subclades between male and female patients in Uzbekistan

Association analysis of different amino acid substitutions, including the two novel Nsp13:A85P and Nsp12:Y479N mutations, no mutation occurred within 39 SARS-CoV-2 genomes have revealed a significant association with disease severity, except for P45L (C27527T) in the ORF7a region. Patients, infected with a SARS-CoV-2 bearing the ORF7a 45P variant found in our study, have developed severe and critical form of the COVID-19. The SARS-CoV-2 bearing 45L amino acid change in the ORF7a was predominantly found in patients with mild symptoms. We found that the ORF7a: 45L has been significantly associated with causing the mild disease symptoms (CMILE OR=0.1; 95% CI 0.01-0.67; p<0,01), while the ORF7a:45P has been significantly associated with

the severe and critical COVID-19 symptoms (CMILE OR=9.1; 95% CI 1.38-84.98; p<0,01) (Table 9).

Table 9. Logistic regression analysis of viral ORF7a:P45L mutation and disease severity.

Mutation	CMLE OR	Lower 95% CI	Upper 95% CI	p-value	p-value (F)
ORF7a: 45L	0.1*	0.01	0.67	0.004	0.03**
ORF7a: 45P	9.1*	1.38	84.98	0.004	0.03**

*Conditional maximum likelihood estimates of Odds Ratio. ** Fisher`s exact test two-tailed p-value

Effect of amino acid substitution in the ORF7a: P45L as well as novel variants of the Nsp13:A85P, and Nsp12: Y479N on protein stability.

To measure protein stability of mutated variants of SARAS-CoV-2 sequenced in our study, we have analyzed the variability of Gibbs free energy of unfolding (ΔΔG) values. We used the diverse prediction models of machine-learning to energy-based force-fields with different software programs (Fig. 11). Analysis of average ΔΔG values, in terms of increased or decreased stability of the ORF7a: P45L and novel variants of the Nsp13: A85P, and Nsp12: Y479N, has revealed a significant decrease of protein stability with an average ΔΔG values of -0.48 (s=0.51), -0.56 (s=1.08), and -1.5; (s=1.26), respectively (Fig. 11). The P45L substitution revealed that non-synonymous Pro to Leu substitution has a deleterious effect, leading to a highly decreased stability of theORF7a protein (Fig.12). Thus, our finding suggests that non - synonymous substitution of amino

acid proline to leucine in the position 45 of the ORF7a protein strongly affects to the functional activity of protein and weakening disease symptoms in comparison with the original coronavirus protein.

Fig. 11. Analysis of different prediction models of the NSP12: Y479N, NSP13: A85P and ORF7a: P45L amino-acid substitution on protein stability. ΔΔG - free energy of unfolding (kcal/mol). The negative and positive predicted ΔΔG values mean the destabilizing and stabilizing effect, respectively [16, 17].

Fig. 12. 3D structure of the ORF7a: P45L substitution effect on protein stability. Dotted line represents hydrogen bonds. Substitution of Pro to Leu (left) results in destabilizing effect due to loss of hydrogen bond.

Discussion

So far, during the pandemic, several factors have had an impact on whether new COVID-19 cases are increasing or declining in particular locations. These factors include the infection prevention policies, human behavior, effectiveness of vaccines over time, changes to the coronavirus itself, and the number of people in population who are vulnerable because of many reasons, including the age, genetic and immune status of hosts, and other social aspects of epidemy.

The study of the global situation showed that the COVID-19 disease pandemic in different countries had a different number of upward periods (incidence peaks), which varied from 0 to 5 waives/incidence, among them the largest number of countries fell in

two incidence peaks [18]. Our study also shows that in Uzbekistan, for the entire period of the COVID_19 disease pandemic, two clear waves were identified. The first wave was thought to be provoked by the spread of the original Wuhan variant [19] in middle of 2020. However, a previous SARS-CoV-2 sample sequencing effort from Uzbekistan, covering the first waive period (November to December of 2020), concluded that the early SARS-CoV-2 infections in our country were distributed from European and Near East countries as a result of international travelling [14] and represented by clades 20B (77,7%) and 19B (22,3%), whereas during the second wave dominant variant was Delta (82%), followed by Alpha (10.3%) and 20A (7.7%) clades (Figs. 6 and 7), comparative phylogenetic tree between SARS-CoV-2 isolates from two waves presented in Fig 13.

Fig.13 Phylogenetic tree of the SARS-CoV-2 isolates distributed in Uzbekistan during the 1-st (2020) and 2-nd (2021) coronavirus pandemic waves. Samples with blue circles are SARS-CoV-2 isolates from the first wave (November 2020), sample with red triangles are from the second wave (mid 2021).

The comparative study of the first wave virus genotypes (Fig 13) implied that the SARS-CoV-2 was distributed to Uzbekistan and not happened directly from China or Wuhan variant but has been spread through other countries after, at least, the first cycle of infection from entered countries. Sequencing based phylogeographic analysis in this study also suggested that there were the multiple independent viral introductions into Uzbekistan from North America, Europe, Africa and Asia [14], which were supported by the evidence of clustered outbreak/community transmission. The second peak occurred in middle of 2021 with the spread of the Delta variant, which eventually became dominant in the world [20] and later in Uzbekistan as shown in our current study. We suggest that the second wave of the SARS-CoV-2 genotype introduction have been mostly occurred

through Russia and Turkey due to the strong socio-economic relationships between Uzbekistan and these countries.

The WGS of isolates our efforts have revealed that an average content of nucleotides was mainly consistent with reference SARS-CoV-2 genome (NC_045512) with slight reduction of G (0.01%) and C (0.05%) nucleotides in comparison to the NC_045512 where G and C accounted for 19.61% and 18.37% respectively [8]. It was found that RNA strand with a high number of C and G bases would form more stable stem-loops than that of a high number of T and A bases. This suggested that the SARS-CoV-2 is more efficient in reproduction than other coronaviruses because less energy is consumed in disrupting the secondary structures formed by its genomic RNA [8]. ~~Because~~ the Delta variant has a higher transmission rate compared to the previous variants, our data suggest that slight reduction of G and C content rate is an evolutionary adaptation of Delta variants for the rapid replication and reproduction in host population.

In our study, among the 12 classes of base substitutions, the C→T transition was dominant (42%). Abundance of C→T transition (40.6%) was observed among the first-wave SARAS-CoV-2 sequencing study from Uzbekistan [14]. Interestingly, we found asymmetry in base changes. For example, the rate of the C→T transition was much higher than that of its reverse T→C substitution (42% and 8%, respectively). Likewise, the rate of G→T transversion was five times higher than that of T→G substitution (16% and 3%, respectively). In this context, our results slightly less percentage from those reported previously by Yi et al. (2021) [21], but we observed similar pattern. Linear changes in the base composition over the time of spread were also observed in Ebola and influenza viruses [22]. Thus, the C→T and G→T asymmetry in the SARS-CoV-2 mutation spectra may be a characteristic of zoonotic RNA viruses recently introduced to human tissues [21].

Each of novel substitutions in the Nsp13: A85P (G16489C) and Nsp12: Y479N (T14875A), found in samples belonging to Delta variant`s subclade 21A (GISAID sample IDs: EPI_ISL_3188967, EPI_ISL_3188979), has been reported for the first time in this study. Both substitutions are located in nonstructural proteins encoded by the ORF1b of a SARS-CoV-2 genome. Nsp13 is a superfamily 1 helicase which acts as motor protein that unwind a double-stranded nucleic acid into the two single-stranded nucleic acid. Nsp12 of coronaviruses encodes RNA-dependent RNA polymerase (RdRp) involved in replication of their genome and for the transcription of their genes [23]. Therefore, novel mutations found in these regions of the SARS-Cov-2 may play an important role in understanding of the COVID-19 disease epidemic.

In the beginning of 2021, the Alpha variant of SARS-CoV-2 was spread rapidly and became dominant in Uzbekistan (unpublished). The situation has changed after the rapid spread of the Delta variant worldwide. In current study, we observed that the Delta variant accounted for 82% among analyzed isolates and became dominant in Uzbekistan in the middle of 2021. Recently, Nextstrain updated its clade designation, partitioning the 21A into the two subclades of 21J and 21I. Clade 21J includes the Delta variant, but it possesses additional ORF1a mutations such as A1306S, P2046L, P2287S, V2930L, T3255I as well as the ORF7b mutation T40I, and the N gene mutation G215C. Among analyzed Delta isolates in our dataset, 44% was designated to 21J subclade, whereas the rest belonged to 21A (56%). There were no samples grouped into 21I subclade in our samples. According to Nextstrain database the Delta 21J variant became predominant worldwide (https://nextstrain.org/ncov/gisaid/global, accessed in December 2021).

The co-occurrence of substitutions in the N: R385K, ORF1a: S944L, H2092Y, L3644F, and ORF1b: H2285Y, identified in our study probably a sign of evolutionary divergence within the subclade 21A; therefore, these mutations require an attention and

should be tracked in further studies. In the S gene, the T95I has occurred in 22% of Delta samples. This finding is in full concordance with previously reported study based on the analysis of 1276 Delta isolates [24], on the other hand, in the same study a distinct substitution G215C in the N genes was found as the Delta plus variants in 58% sequences samples, whereas in current study this substitution was identified in 46.8% of Delta isolates.

Analysis of predicted Nsp12 and Nsp13 stability due to the respective Tyr479Asn and Ala85Pro substitutions resulted in decrease of an average ΔΔG values of these proteins for minus 1.5; and minus 0.56, respectively (Fig. 11). Decreased stability and low substitution frequency in these core proteins of the SARS-CoV-2, found in our study, suggested that the Nsp13:A85P and Nsp12:Y479N mutations may not have an evolutionary advantage for SARS-CoV-2. Nevertheless, it was reported that the NSP13 downregulates interferon production and signaling as well as NF-κB promoter signaling by limiting the TBK1 and IRF3 activation [25, 26], whereas the Nsp12 attenuates type I interferon production by inhibiting IRF3 nuclear translocation [27]. These implies that the Nsp13:A85P and Nsp12:Y479N substitutions may have an impact on primary interferon suppression in the host cells and can antagonize host antiviral innate immunity. Therefore, further research and monitoring of the spread of these mutations is required.

The statistically significant gender distribution bias among the subclades 21A and 21J, found in sequenced WGS in our study, could be a population specific gender susceptibility to the SARS-CoV-2 clades and underlies a need for further comparative studies in other populations. Previous global GISAID-derived metadata analysis also reported statistically significant gender bias among several SARS-CoV-2 clades [28]. Thus, there is a gender specific susceptibility to SARS-CoV-2 variants worldwide and

further studies needed to be conducted to elucidate the possible genetic mechanisms of this phenomenon.

The association of ORF7a: P45L mutation with disease severity in Uzbekistan, reported for the first time herein, is in concordance with previous research. that the stabilizing mutation in the ORF7a of a SARS-CoV-2 was associated with the increased severity and lethality in a group of Romanian patients, despite a lower viral copy number and a lower number of associated comorbidities [29].

The ORF7a protein is thought to be a type I transmembrane protein. The structure of the SARS-CoV protein ORF7a, shows similarities to the immunoglobulin-like (Ig-like) fold with some features resembling those of the DI domain of ICAM-1 and suggests a binding activity to integrin I domains [30]. It is known that Ig-like domain-containing proteins play vital roles in mediating macromolecular interactions in the immune system. Since SARS-CoV-2 ORF7a, similar to SARS-CoV ORF7a, it is predicted to be a member of the Ig-like domain superfamily [31-33]. ORF7a may play a significant role in the clinical severity of COVID-19 [34]. Recent study demonstrated that SARS-CoV-2 ORF7a coincubation with CD14+ monocytes *ex vivo* triggered a decrease in HLA-DR/DP/DQ expression levels and upregulated significant production of proinflammatory cytokines, including IL-6, IL-1β, IL-8, and TNF-α. Thus, it demonstrates that the SARS-CoV-2 ORF7a is an immunomodulating factor for immune cell binding and triggers dramatic inflammatory responses [33]. This forms the basis of a likely mechanism through which ORF7a mediates the potentially fatal cytokine storm progression in COVID-19 patients, indicating that ORF7a may be a key viral factor for disease severity.

The ORF7a: P45L mutation of the Delta became dominant in Russia [35]. It was also found in the Delta variants isolates from India [36], and in the Omicron samples from South Africa (EPI_ISL_6647958). We suggest that the 45L mutation in the ORF7a is the

next step in the evolution of the coronavirus towards a decrease in the severity of the disease, since the main guarantee of the existence and spread of the virus is not the death of the host, but the evolution towards an increase in infectivity.

Materials and methods

Samples collection

The SARS-CoV-2 strains were isolated from nasopharyngeal swab samples (Huachenyang Technology, Shenzhen, China) of SARS-CoV-2 qPCR positive (Ct≤28) patients with mild, moderate, and severe symptoms who were being treated at the State Hospital Zangiota-1 located in Tashkent, Uzbekistan, during July 2021. The study included 20 male and 19 female patients, a total of 39 samples with average ages of 55,21 (SD=14,9; Table 1). Ethical approval for this study was obtained from Ethics Committee of Center for Advanced Technologies under Ministry of Innovative development (Approval Date: May 5, 2021; Approval Number CAT-EC-2021/05-1). Samples collected were anonymized and distinct ID numbers were assigned to each, only keeping age and biological sex for the downstream analysis and reporting purposes. Because of anonymity of the collected data, a voluntary participation condition and non-invasiveness of sequencing experiment, with clear explanation of the purpose of sample collection to each participant, we received their verbal consent. All the experiments were carried out in accordance with the relevant guidelines and regulations.

Table 10. Age and gender distribution of studied patients.

	Mean Age (SD)	Number of patients
Male	50 (14,7)	20

Female	60.7 (13,2)	19
Total (M+F)	55.21 (14,9)	39

Data collection

Epidemiological, clinical, and disease severity data were extracted from electronic medical records using a standardized data collection form, kindly provided by State Hospital Zangiota-1 located in Tashkent, Uzbekistan. All data records were matched with previously assigned sample IDs and used as anonymized dataset for downstream analyses.

SARS-CoV-2 nucleic acid isolation

Total nucleic acid was extracted using the QIAseq DIRECT SARS-CoV-2 (Qiagen GmbH, Hilden, Germany). RNA quantity was evaluated through the Qubit RNA HS assays kits (Life Technologies, Carlsbad, California, USA) on Qubit® 2.0 Fluorometer (Life Technologies, Carlsbad, California, USA) according to manufacturer’s manual. The presence of SARS-CoV-2 in the purified RNA samples for the downstream steps was estimated by fluorescence detection of RdRp and N genes using Biotest SARS-CoV-2 RT-qPCR Kit (Biotest Lab LLC, Tashkent, Uzbekistan) according to manufacturer’s protocol on the QuantStudio™ 5 Real-Time PCR System (Applied Biosystems, Foster City, USA).

Next-Generation Sequencing

Whole genome amplification of the SARS-CoV-2 was performed using the CleanPlex® SARS-CoV-2 Research and Surveillance NGS Panel (Paragon Genomics Inc., Hayward, CA, USA). Briefly, cDNA was generated from previously extracted RNA using RT Primer

Mix DP followed by RT reaction purification from RNA using CleanMag® Magnetic Beads (Paragon Genomics Inc., Hayward, CA, USA). Then, a multiplex PCR (mPCR) reaction using target-specific primers to amplify the entire SARS-CoV-2 genome was performed, using a 2-pool design, followed by digestion and post-digestion purification, and second mPCR reaction. This was to amplify and add sample-level i5 and i7 primers into the generated libraries for the Illumina sequencing platforms (Illumina, San Diego, CA, USA). Finally, the library was purified using CleanMag® Magnetic Beads (Paragon Genomics Inc., Hayward, CA, USA). Libraries were evaluated by gel-electrophoresis and considered for sequencing when a fragment size ~ 275 bp was obtained and the final concentration was above 2.0 ng/µl, measured on Qubit™ with dsDNA HS Assay Kit (Thermo Fisher Scientific, Waltham, MA, USA).

After confirmation of the library quality, libraries were normalized to 10 nM and samples with unique index combinations were pooled in equimolar ratios to reach the recommended final concentration of 4 nM for sequencing. After a further quantification with Qubit dsDNA HS Assay Kit (Thermo Fisher Scientific, Waltham, MA, USA), pooled libraries were prepared for the sequencing, following the Standard Normalization protocol on MiSeq System Denature and Dilute Libraries Guide (Illumina, San Diego, CA, USA) with a final denaturation and dilution to 11 pM. Sequencing was carried-out on a MiSeq instrument (Illumina, San Diego, CA, USA) with Reagent Kit v3, using 20 pM PhiX control spike-in of 5% for low-diversity libraries, setting 2 x 150 cycles, and generating paired-end reads.

Genomic analyses, variants assessments and phylogeny

NGS raw data (FASTQ files) were generated from MiSeq Local Run Manager (Illumina, San Diego, CA, USA) and uploaded on the SOPHiA DDM platform (SOPHiA Genetics, Lausanne, Switzerland) for the external quality check, trimming of adaptors, variant call

review, re-alignment of indels, quality measurements, and determination of the consensus genome by mapping to reference sequence MN908947 (NC 045512.2). For this, we have used a proprietary design pipeline to cover the entire genome.

The public database GISAID [15] was used for the BLAST searches and for mutation analysis. The Nextclade Web tool v.1.11.1 [37] was used to compare study sequences to SARS-CoV-2 reference sequences, assign them to clades, and determine their position within the SARS-CoV-2 phylogenetic tree.

Variant calling, and mutation identification was also additionally performed by using Genome Detective Coronavirus Typing Tool [38], CoV-GLUE v.1.1.108 [39] and COVID-19 genome annotator (http://giorgilab.unibo.it/coronannotator/).

The full-length genomic sequences of 39 coronaviruses were aligned using the L-INS-i method of MAFFT v7.310 [40]. Phylogenetic tree of 39 samples, reference genome and genomes of variants constructed by the Neighbor-Joining method [41] was performed using MEGA X software [42]. The bootstrap consensus tree inferred from 10000 replicates is taken to represent the evolutionary history of the taxa analyzed. Branches corresponding to partitions reproduced in less than 50% bootstrap replicates are collapsed. The evolutionary distances were computed using the Maximum Composite Likelihood method. Codon positions included were 1st+2nd+3rd+Noncoding. All ambiguous positions were removed for each sequence pair (pairwise deletion option). The phylogenetic trees were visualized by MEGA X [42] and FigTree v1.4.4 software (http://tree.bio.ed.ac.uk/software/figtree/).

Data Availability

Nucleotide sequences of SARS-CoV-2 isolates from Uzbekistan were submitted to GISAID on 2.08.2021 and available for registered users at https://www.epicov.org/,

accession IDs from EPI_ISL_3188963 to EPI_ISL_3189001. The full sequence reads and genome annotation data used and/or analyzed in this study are also available in the S1 and S2 File.

Statistical analysis

Summary statistics and the distribution of the primary data were visualized using BoxPlotR [43]. For association analysis, patients with mild and moderate disease severity were pooled into one "non-severe" category and compared with the "severe" (severe-critical) patient group. The association between viral genotypes and disease severity was investigated using the Fisher exact test and odds ratio (OR) calculation via a 2×2 contingency table. Comparisons were made between our phylogenetic clades [e.g., a number of patients, infected with a SARS-CoV-2 from the clade 21A (or defined SNPs) versus the number of patients, not infected with a SARS-CoV-2 from the clade 21J (or defined SNPs] and the different categories of disease severity (e.g., a number of patients with severe disease versus a number of patients without severe disease).

Protein stability prediction

Prediction the increased or decreased stability of protein upon amino-acid substitution was done by the estimation of difference in Gibbs free energy of unfolding (ΔΔG value in kcal/mol) between the mutated and wild-type proteins ($\Delta\Delta G = \Delta G_{mutant} - \Delta G_{wild\text{-}type}$) [16, 44]. Although many prediction tools are available, the conflicting prediction results from different tools could cause confusion to users. In order to avoid such bias in predicting proteins stability, several programs have been used in our analyses, which exploited the diverse prediction models from machine-learning to energy-based force-fields [45], and the average ΔΔG value was calculated for each protein.

References

1. COVID-19 Map - Johns Hopkins Coronavirus Resource Center. [cited 16 Sep 2021]. Available: https://coronavirus.jhu.edu/map.html

2. Mercatelli D, Giorgi FM. Geographic and Genomic Distribution of SARS-CoV-2 Mutations. Front Microbiol. 2020;11: 1800. doi:10.3389/fmicb.2020.01800

3. Wang M, Fu A, Hu B, Tong Y, Liu R, Liu Z, et al. Virus Detection: Nanopore Targeted Sequencing for the Accurate and Comprehensive Detection of SARS-CoV-2 and Other Respiratory Viruses (Small 32/2020). Small. 2020;16. doi:10.1002/smll.202070175

4. Lauring AS, Hodcroft EB. Genetic Variants of SARS-CoV-2—What Do They Mean? JAMA. 2021;325: 529–531. doi:10.1001/jama.2020.27124

5. Lau BT, Pavlichin D, Hooker AC, Almeda A, Shin G, Chen J, et al. Profiling SARS-CoV-2 mutation fingerprints that range from the viral pangenome to individual infection quasispecies. medRxiv. 2020; 2020.11.02.20224816. doi:10.1101/2020.11.02.20224816

6. Mousavizadeh L, Ghasemi S. Genotype and phenotype of COVID-19: Their roles in pathogenesis. J Microbiol Immunol Infect. 2021;54: 159–163. doi:10.1016/j.jmii.2020.03.022

7. Yin C. Genotyping coronavirus SARS-CoV-2: methods and implications. Genomics. 2020;112: 3588–3596. doi:10.1016/j.ygeno.2020.04.016

8. Wang Y, Mao J-M, Wang G-D, Luo Z-P, Yang L, Yao Q, et al. Human SARS-CoV-2 has evolved to reduce CG dinucleotide in its open reading frames. Sci Rep. 2020;10: 12331. doi:10.1038/s41598-020-69342-y

9. Harvey WT, Carabelli AM, Jackson B, Gupta RK, Thomson EC, Harrison EM, et al. SARS-CoV-2 variants, spike mutations and immune escape. Nat Rev Microbiol. 2021;19: 409–424. doi:10.1038/s41579-021-00573-0

10. Dao TL, Hoang VT, Colson P, Lagier JC, Million M, Raoult D, et al. SARS-CoV-2 Infectivity and Severity of COVID-19 According to SARS-CoV-2 Variants: Current Evidence. J Clin Med. 2021;10: 2635. doi:10.3390/jcm10122635

11. Wu A, Niu P, Wang L, Zhou H, Zhao X, Wang W, et al. Mutations, Recombination and Insertion in the Evolution of 2019-nCoV. bioRxiv; 2020. p. 2020.02.29.971101. doi:10.1101/2020.02.29.971101

12. Lu R, Zhao X, Li J, Niu P, Yang B, Wu H, et al. Genomic characterisation and epidemiology of 2019 novel coronavirus: implications for virus origins and receptor binding. The Lancet. 2020;395: 565–574. doi:10.1016/S0140-6736(20)30251-8

13. Rahnavard A, Dawson T, Clement R, Stearrett N, Pérez-Losada M, Crandall KA. Epidemiological associations with genomic variation in SARS-CoV-2. Sci Rep. 2021;11: 23023. doi:10.1038/s41598-021-02548-w

14. Ayubov MS, Buriev ZT, Mirzakhmedov MK, Yusupov AN, Usmanov DE, Shermatov SE, et al. Profiling of the most reliable mutations from sequenced SARS-

CoV-2 genomes scattered in Uzbekistan. bioRxiv. 2022; 2022.02.10.479714. doi:10.1101/2022.02.10.479714

15. Shu Y, McCauley J. GISAID: Global initiative on sharing all influenza data – from vision to reality. Eurosurveillance. 2017;22: 30494. doi:10.2807/1560-7917.ES.2017.22.13.30494

16. Sanavia T, Birolo G, Montanucci L, Turina P, Capriotti E, Fariselli P. Limitations and challenges in protein stability prediction upon genome variations: towards future applications in precision medicine. Comput Struct Biotechnol J. 2020;18: 1968–1979. doi:10.1016/j.csbj.2020.07.011

17. Parthiban V, Gromiha MM, Schomburg D. CUPSAT: prediction of protein stability upon point mutations. Nucleic Acids Res. 2006;34: W239–W242. doi:10.1093/nar/gkl190

18. Zhang SX, Arroyo Marioli F, Gao R, Wang S. A Second Wave? What Do People Mean by COVID Waves? – A Working Definition of Epidemic Waves. Risk Manag Healthc Policy. 2021;14: 3775–3782. doi:10.2147/RMHP.S326051

19. Wu F, Zhao S, Yu B, Chen Y-M, Wang W, Song Z-G, et al. A new coronavirus associated with human respiratory disease in China. Nature. 2020;579: 265–269. doi:10.1038/s41586-020-2008-3

20. Cherian S, Potdar V, Jadhav S, Yadav P, Gupta N, Das M, et al. SARS-CoV-2 Spike Mutations, L452R, T478K, E484Q and P681R, in the Second Wave of COVID-19 in Maharashtra, India. Microorganisms. 2021;9: 1542. doi:10.3390/microorganisms9071542

21. Yi K, Kim SY, Bleazard T, Kim T, Youk J, Ju YS. Mutational spectrum of SARS-CoV-2 during the global pandemic. Exp Mol Med. 2021;53: 1229–1237. doi:10.1038/s12276-021-00658-z

22. Wada Y, Wada K, Iwasaki Y, Kanaya S, Ikemura T. Directional and reoccurring sequence change in zoonotic RNA virus genomes visualized by time-series word count. Sci Rep. 2016;6: 36197. doi:10.1038/srep36197

23. Snijder EJ, Decroly E, Ziebuhr J. Chapter Three - The Nonstructural Proteins Directing Coronavirus RNA Synthesis and Processing. In: Ziebuhr J, editor. Advances in Virus Research. Academic Press; 2016. pp. 59–126. doi:10.1016/bs.aivir.2016.08.008

24. Kannan SR, Spratt AN, Cohen AR, Naqvi SH, Chand HS, Quinn TP, et al. Evolutionary analysis of the Delta and Delta Plus variants of the SARS-CoV-2 viruses. J Autoimmun. 2021;124: 102715. doi:10.1016/j.jaut.2021.102715

25. Yuen C-K, Lam J-Y, Wong W-M, Mak L-F, Wang X, Chu H, et al. SARS-CoV-2 nsp13, nsp14, nsp15 and orf6 function as potent interferon antagonists. Emerg Microbes Infect. 2020;9: 1418–1428. doi:10.1080/22221751.2020.1780953

26. Vazquez C, Swanson SE, Negatu SG, Dittmar M, Miller J, Ramage HR, et al. SARS-CoV-2 viral proteins NSP1 and NSP13 inhibit interferon activation through

distinct mechanisms. PLOS ONE. 2021;16: e0253089. doi:10.1371/journal.pone.0253089

27. Wang W, Zhou Z, Xiao X, Tian Z, Dong X, Wang C, et al. SARS-CoV-2 nsp12 attenuates type I interferon production by inhibiting IRF3 nuclear translocation. Cell Mol Immunol. 2021;18: 945–953. doi:10.1038/s41423-020-00619-y

28. Hamed SM, Elkhatib WF, Khairalla AS, Noreddin AM. Global dynamics of SARS-CoV-2 clades and their relation to COVID-19 epidemiology. Sci Rep. 2021;11: 8435. doi:10.1038/s41598-021-87713-x

29. Lobiuc A, Șterbuleac D, Sturdza O, Dimian M, Covasa M. A Conservative Replacement in the Transmembrane Domain of SARS-CoV-2 ORF7a as a Putative Risk Factor in COVID-19. Biology. 2021;10: 1276. doi:10.3390/biology10121276

30. Hänel K, Stangler T, Stoldt M, Willbold D. Solution structure of the X4 protein coded by the SARS related coronavirus reveals an immunoglobulin like fold and suggests a binding activity to integrin I domains. J Biomed Sci. 2006;13: 281–293. doi:10.1007/s11373-005-9043-9

31. Nelson CA, Pekosz A, Lee CA, Diamond MS, Fremont DH. Structure and Intracellular Targeting of the SARS-Coronavirus Orf7a Accessory Protein. Structure. 2005;13: 75–85. doi:10.1016/j.str.2004.10.010

32. Tan Y, Schneider T, Leong M, Aravind L, Zhang D. Novel Immunoglobulin Domain Proteins Provide Insights into Evolution and Pathogenesis of SARS-CoV-2-Related Viruses. mBio. 2020 [cited 21 Feb 2022]. doi:10.1128/mBio.00760-20

33. Zhou Z, Huang C, Zhou Z, Huang Z, Su L, Kang S, et al. Structural insight reveals SARS-CoV-2 ORF7a as an immunomodulating factor for human CD14+ monocytes. iScience. 2021;24: 102187. doi:10.1016/j.isci.2021.102187

34. Su C-M, Wang L, Yoo D. Activation of NF-κB and induction of proinflammatory cytokine expressions mediated by ORF7a protein of SARS-CoV-2. Sci Rep. 2021;11: 13464. doi:10.1038/s41598-021-92941-2

35. Klink GV, Safina K, Nabieva E, Shvyrev N, Garushyants S, Alekseeva E, et al. The rise and spread of the SARS-CoV-2 AY.122 lineage in Russia. medRxiv. 2021; 2021.12.02.21267168. doi:10.1101/2021.12.02.21267168

36. Das JK, Sengupta A, Choudhury PP, Roy S. Characterizing genomic variants and mutations in SARS-CoV-2 proteins from Indian isolates. Gene Rep. 2021;25: 101044. doi:10.1016/j.genrep.2021.101044

37. Hadfield J, Megill C, Bell SM, Huddleston J, Potter B, Callender C, et al. Nextstrain: real-time tracking of pathogen evolution. Bioinformatics. 2018;34: 4121–4123. doi:10.1093/bioinformatics/bty407

38. Cleemput S, Dumon W, Fonseca V, Abdool Karim W, Giovanetti M, Alcantara LC, et al. Genome Detective Coronavirus Typing Tool for rapid identification and characterization of novel coronavirus genomes. Bioinformatics. 2020;36: 3552–3555. doi:10.1093/bioinformatics/btaa145

39. Singer J, Gifford R, Cotten M, Robertson D. CoV-GLUE: A Web Application for Tracking SARS-CoV-2 Genomic Variation. 2020. doi:10.20944/preprints202006.0225.v1

40. Katoh K, Standley DM. MAFFT Multiple Sequence Alignment Software Version 7: Improvements in Performance and Usability. Mol Biol Evol. 2013;30: 772–780. doi:10.1093/molbev/mst010

41. Saitou N, Nei M. The neighbor-joining method: a new method for reconstructing phylogenetic trees. Mol Biol Evol. 1987;4: 406–425. doi:10.1093/oxfordjournals.molbev.a040454

42. Kumar S, Stecher G, Li M, Knyaz C, Tamura K. MEGA X: Molecular Evolutionary Genetics Analysis across Computing Platforms. Mol Biol Evol. 2018;35: 1547–1549. doi:10.1093/molbev/msy096

43. Spitzer M, Wildenhain J, Rappsilber J, Tyers M. BoxPlotR: a web tool for generation of box plots. Nat Methods. 2014;11: 121–122. doi:10.1038/nmeth.2811

44. Tokuriki N, Stricher F, Serrano L, Tawfik DS. How Protein Stability and New Functions Trade Off. PLOS Comput Biol. 2008;4: e1000002. doi:10.1371/journal.pcbi.1000002

45. Caldararu O, Blundell TL, Kepp KP. A base measure of precision for protein stability predictors: structural sensitivity. BMC Bioinformatics. 2021;22: 88. doi:10.1186/s12859-021-04030-w

Acknowledgments

We thank the doctors and nurses of the COVID-19 clinics at the State Hospital Zangiota-1 Tashkent, Uzbekistan for their help in collection of the samples and disease data from symptomatic patients.

Conflict of Interests

The authors declare that there are no conflicts of interest.

Author Contributions

Conceptualization: Alisher Abdullaev, Shakhlo U. Turdikulova

Data curation: Alisher Abdullaev, Dilbar Dalimova, Vladimir Tsoy

Funding acquisition: Shakhlo U. Turdikulova**,** Dilbar Dalimova

Investigation: Alisher Abdullaev, Zebiniso Mirakbarova, Shakhnoza Ibragimova, Vladimir Tsoy, Sharofiddin Nuriddinov

Methodology: Alisher Abdullaev, Dilbar Dalimova, Shakhlo U. Turdikulova

Visualization: Alisher Abdullaev

Supervision: Shakhlo U. Turdikulova, Ibrokhim Y. Abdurakhmonov

Writing – original draft: Alisher Abdullaev

Writing – review & editing: Ibrokhim Y. Abdurakhmonov, Alisher Abdullaev, Shakhlo U. Turdikulova

Funding

This study has been supported by the research grant from the Ministry of Innovative Development, Republic of Uzbekistan (Research Grant number: А-ИРВ-2021-125). The funders had no role in study design, data collection and analysis, decision to publish, or preparation of the manuscript.

Figure 1

Click here to access/download;Figure;Fig1.tiff

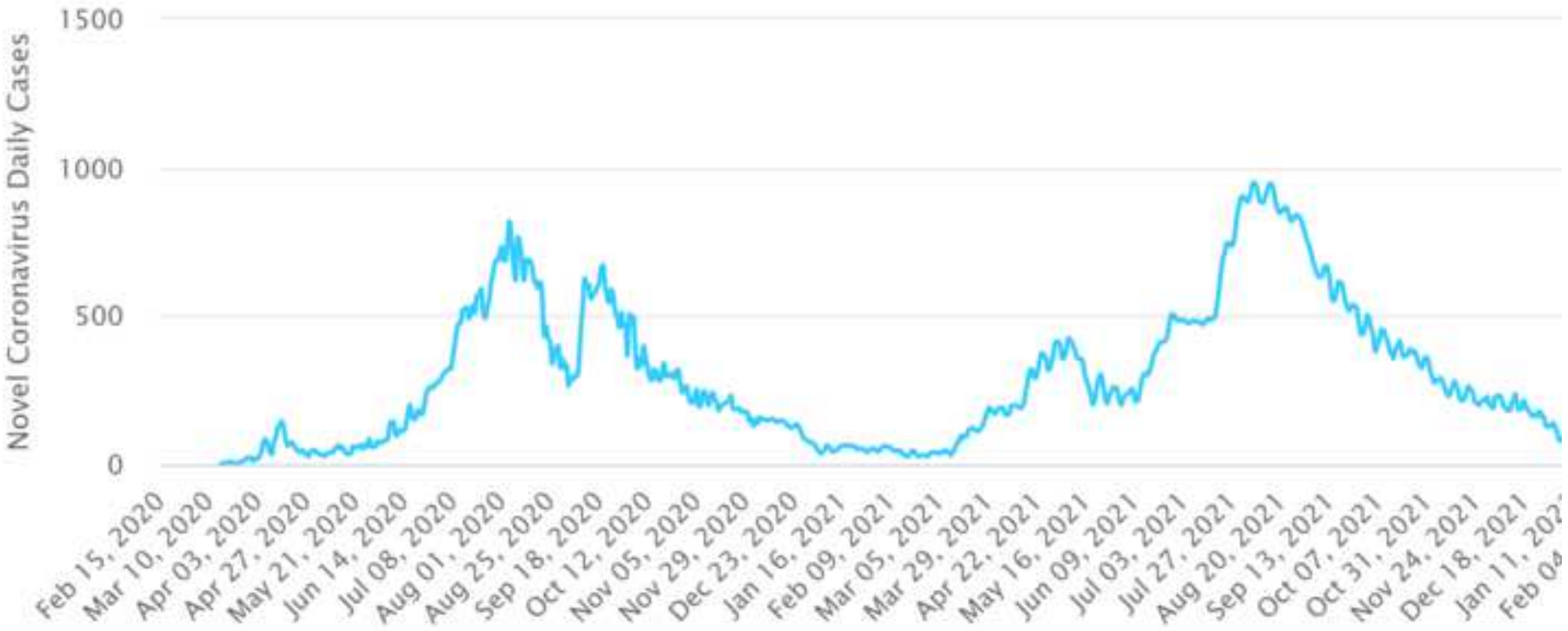

Figure 2

Click here to access/download;Figure;Fig2.tiff

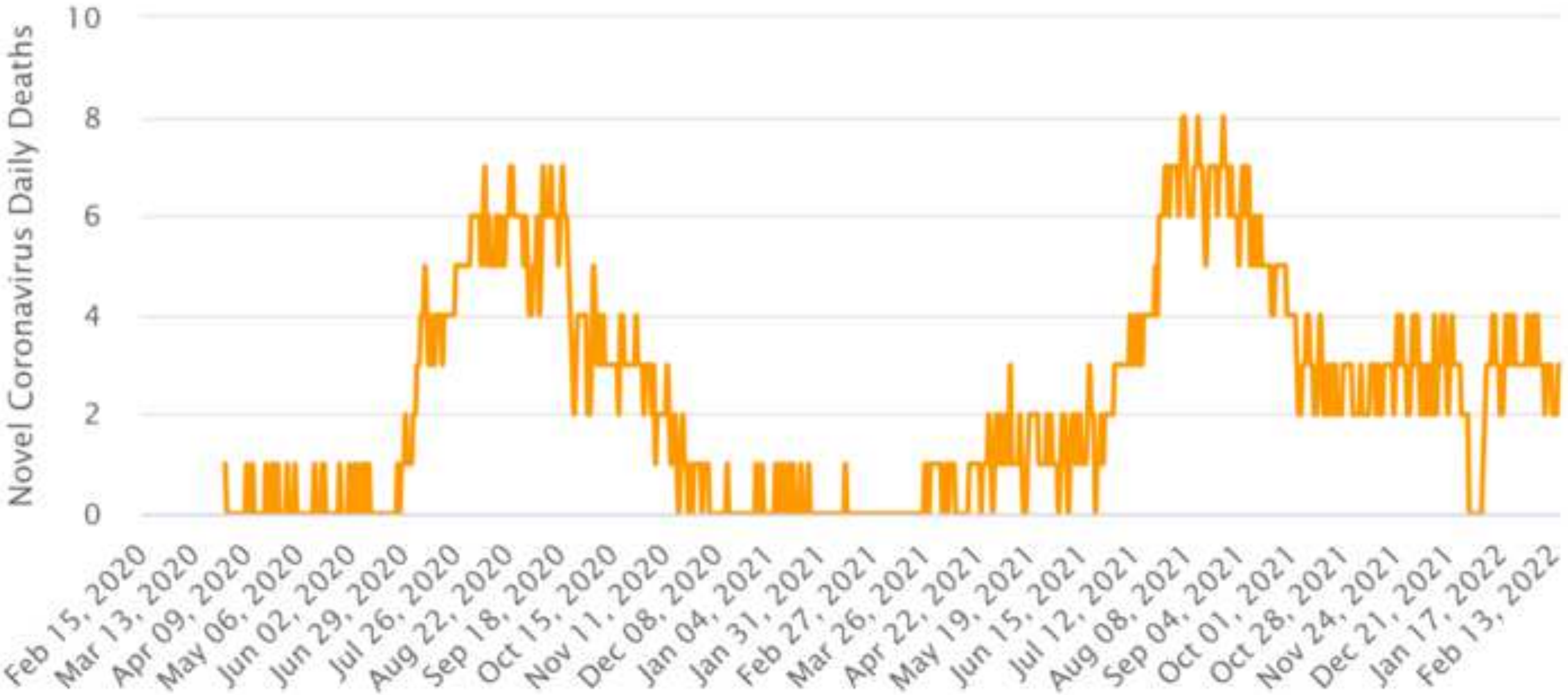

Figure 3

Click here to access/download;Figure;Fig3.tif

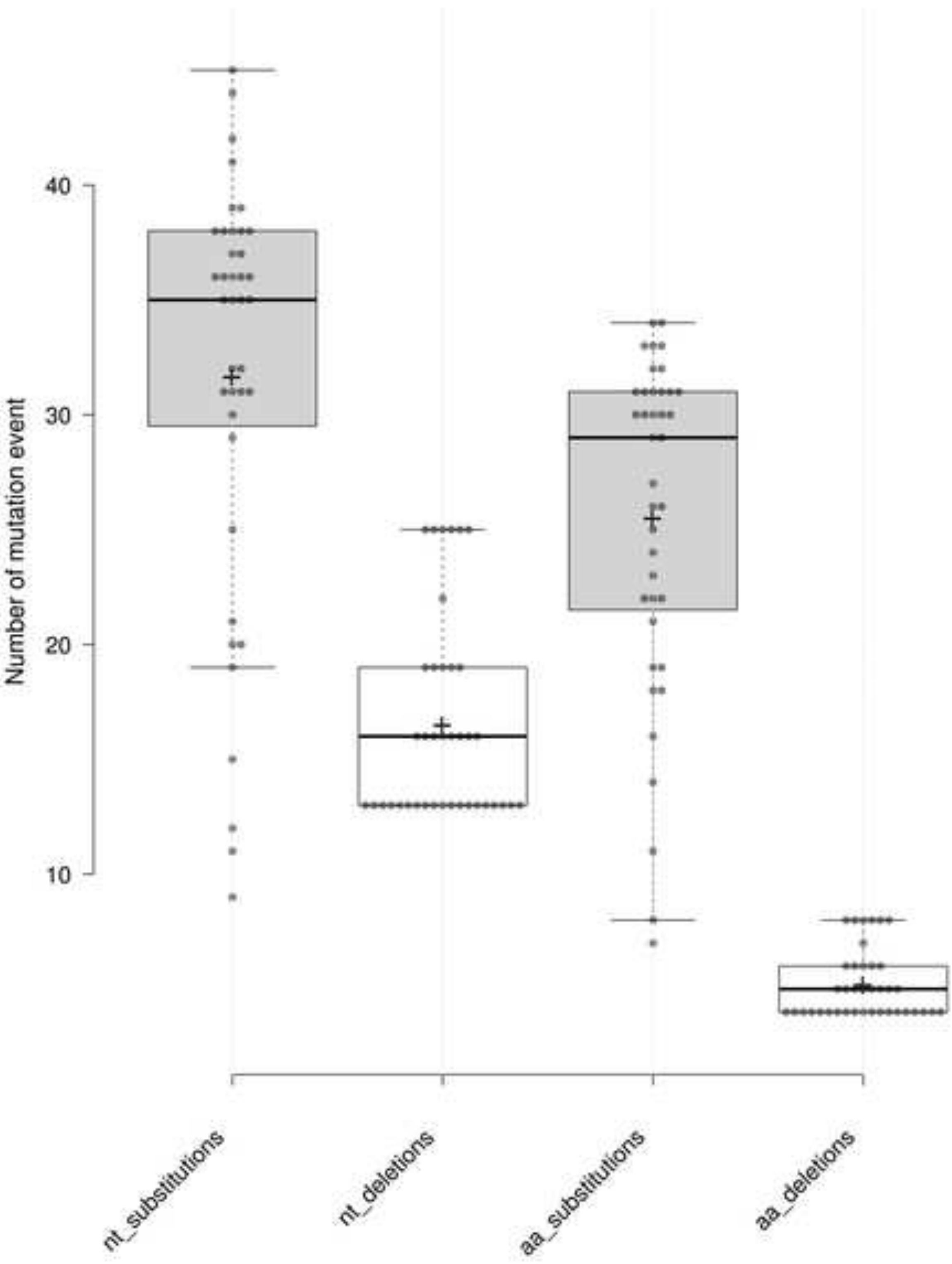

Figure 4

Click here to access/download;Figure;Fig4.tiff

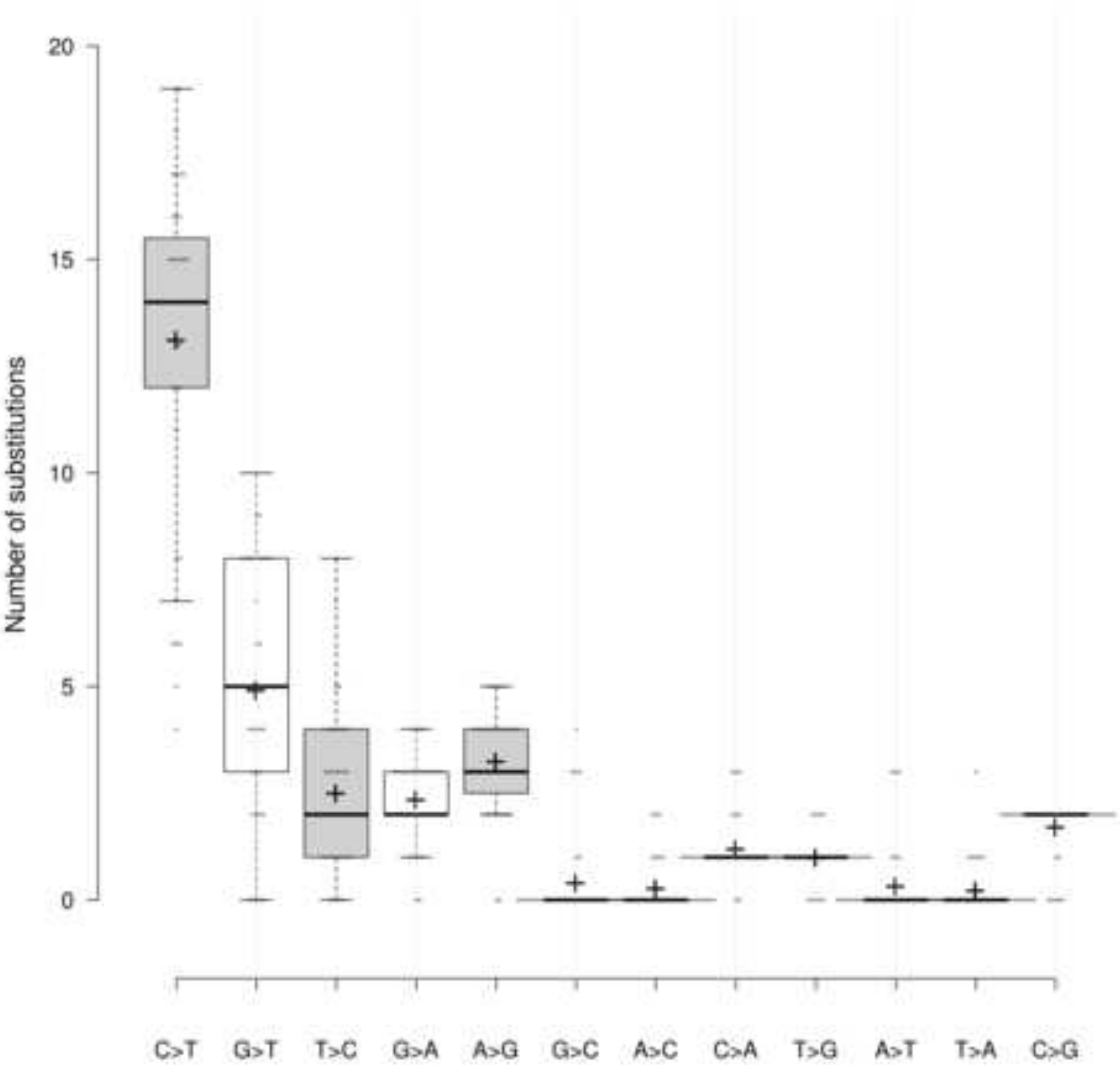

Figure 5

Click here to access/download;Figure;Fig5.tiff

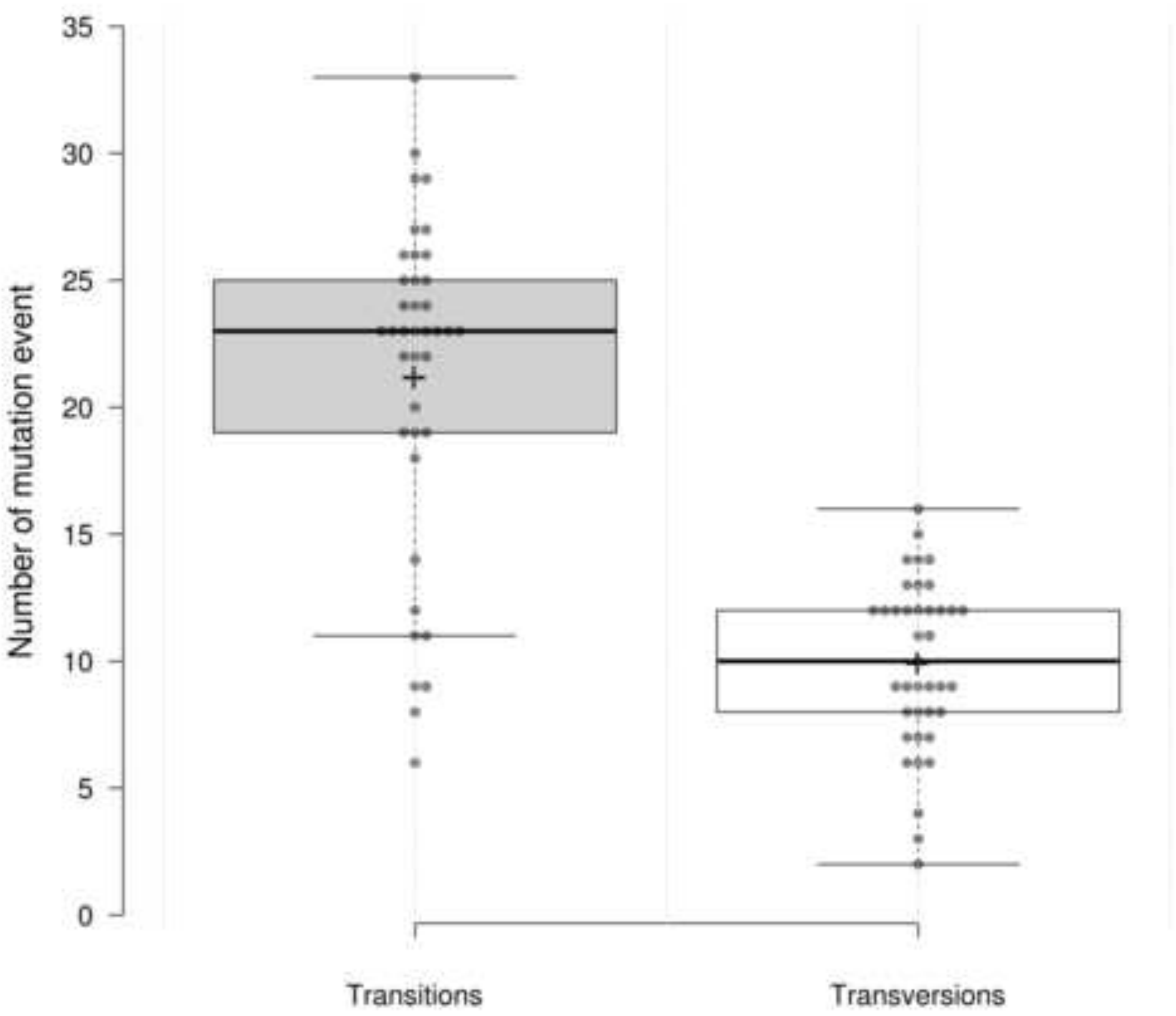

Figure 6

Click here to access/download;Figure;Fig6.tif

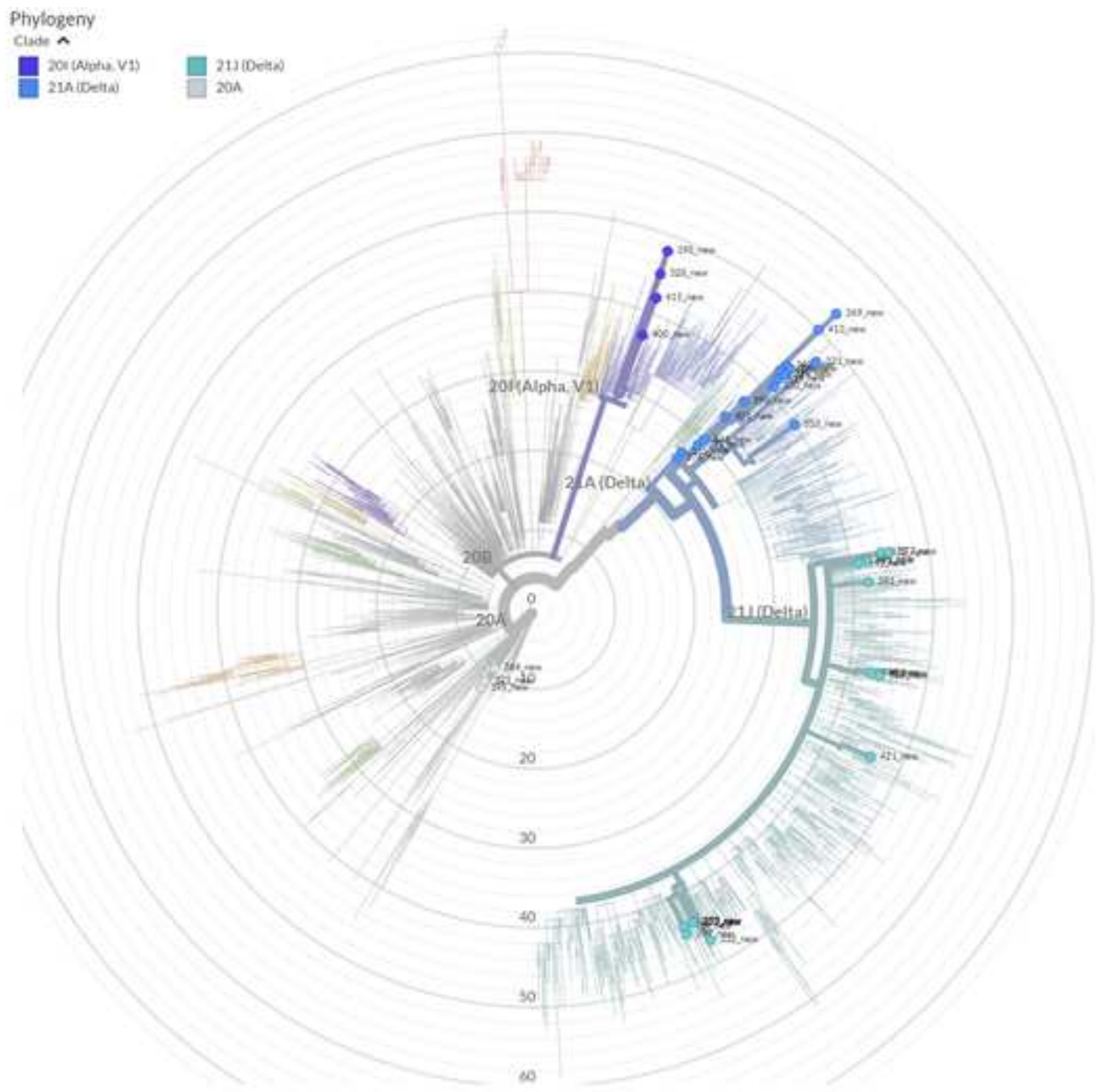

Figure 7

Click here to access/download;Figure;Fig7-2021_boostrap.tif

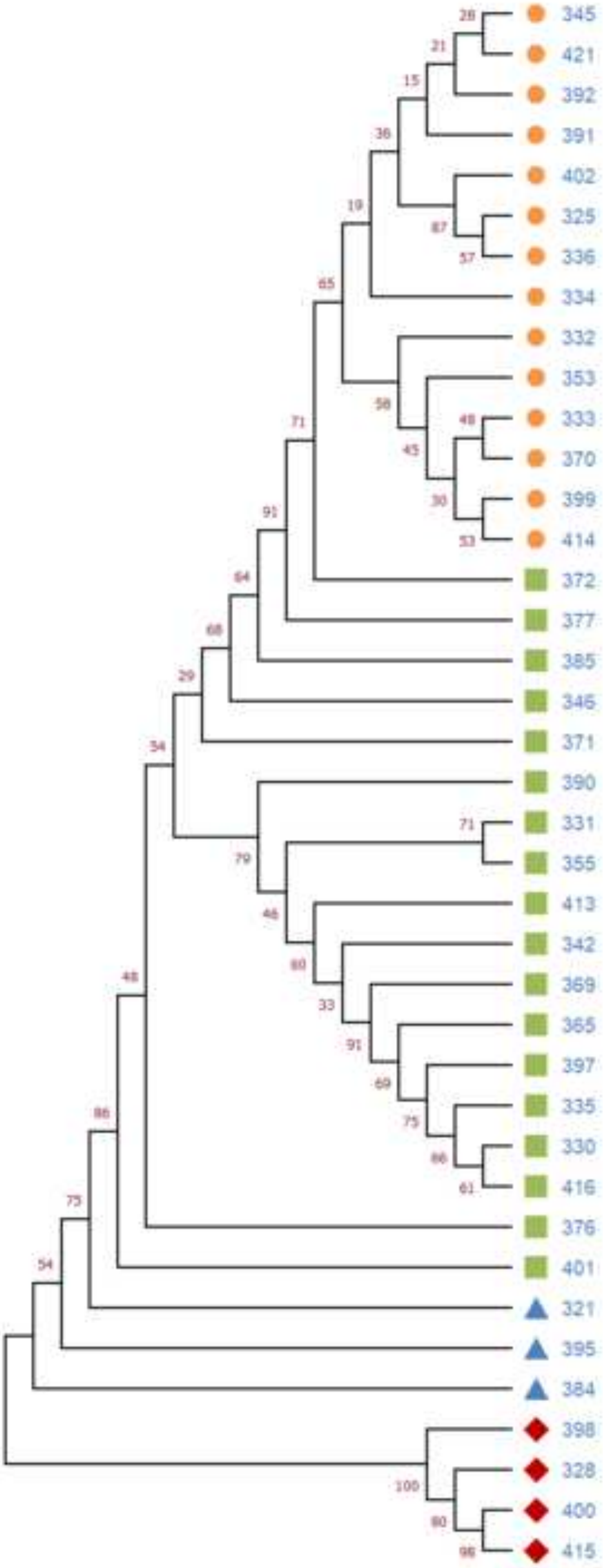

Figure 8

Click here to access/download;Figure;Fig8.tif

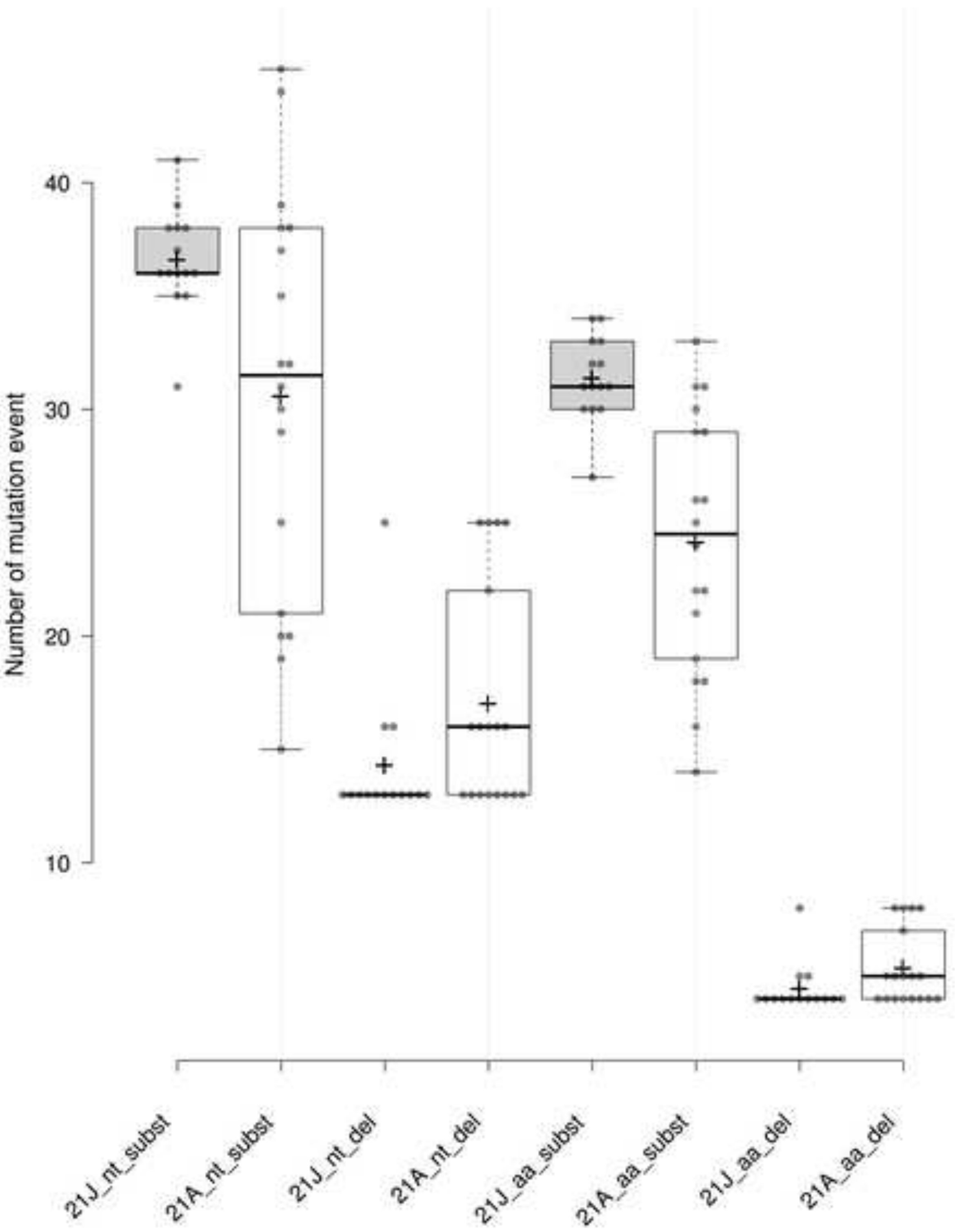

Figure 9

Click here to access/download;Figure;Fig9.tiff

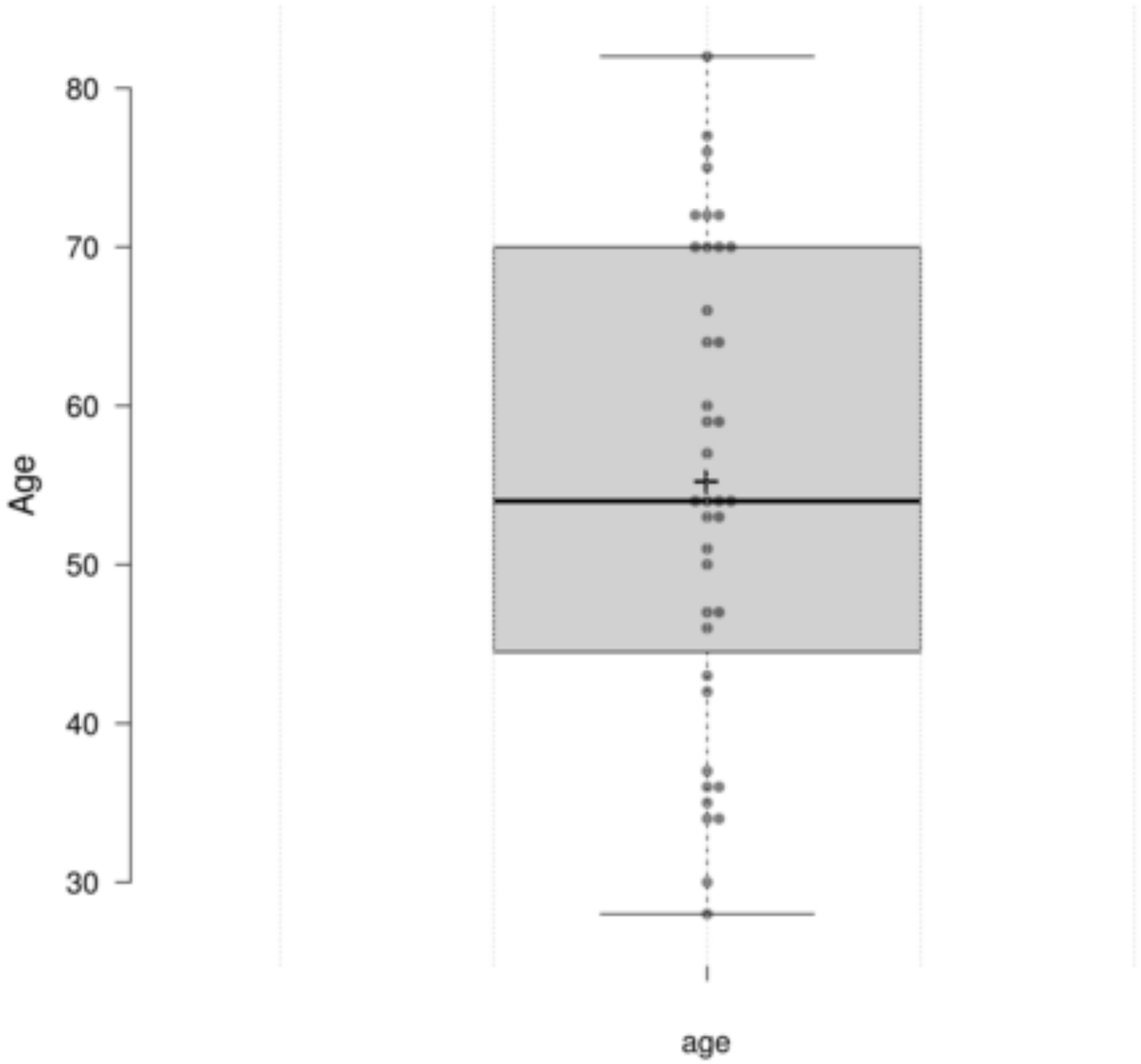

Figure 10

Click here to access/download;Figure;Fig10.tif

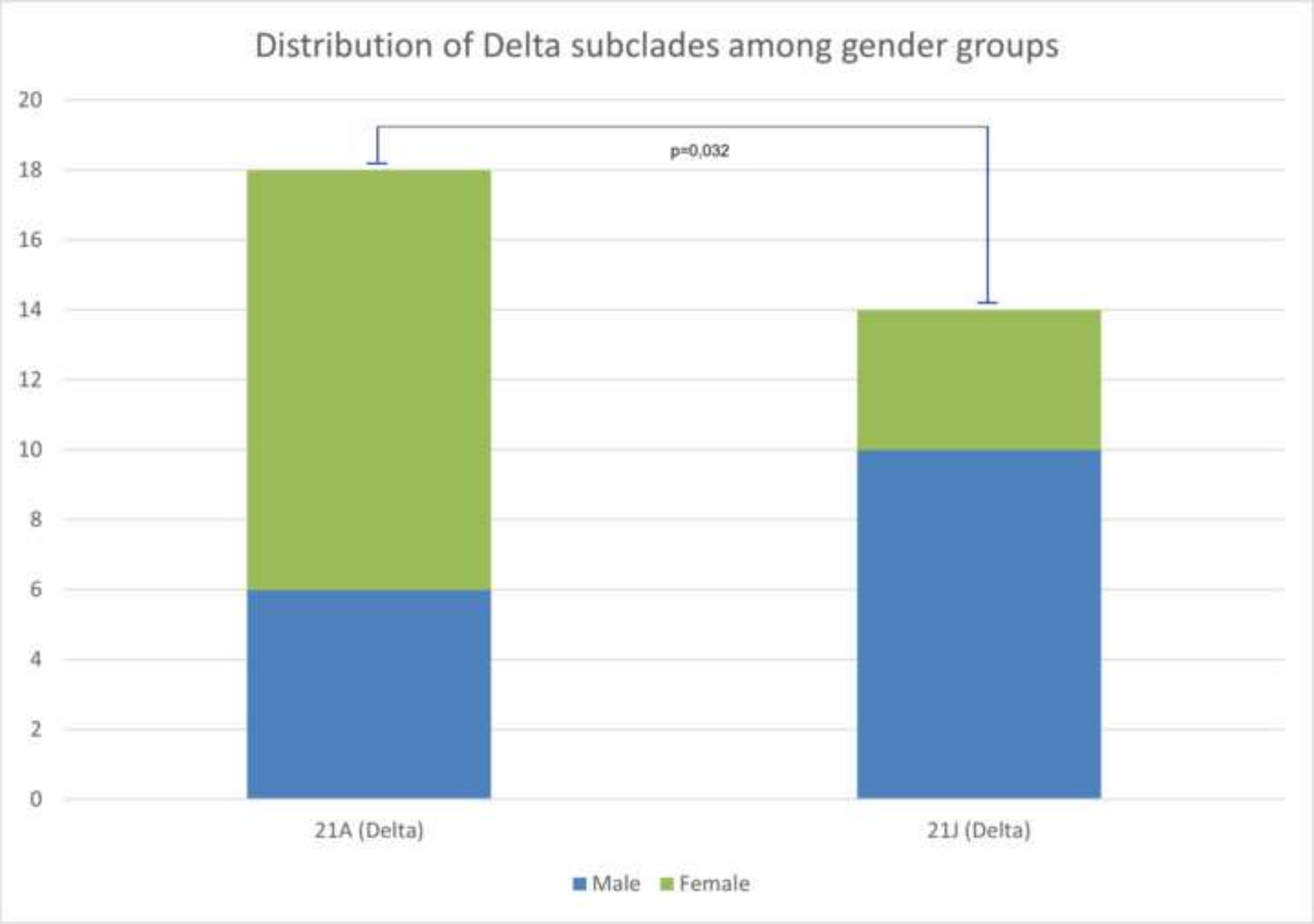

Figure 11

Click here to access/download;Figure;Fig11.tif

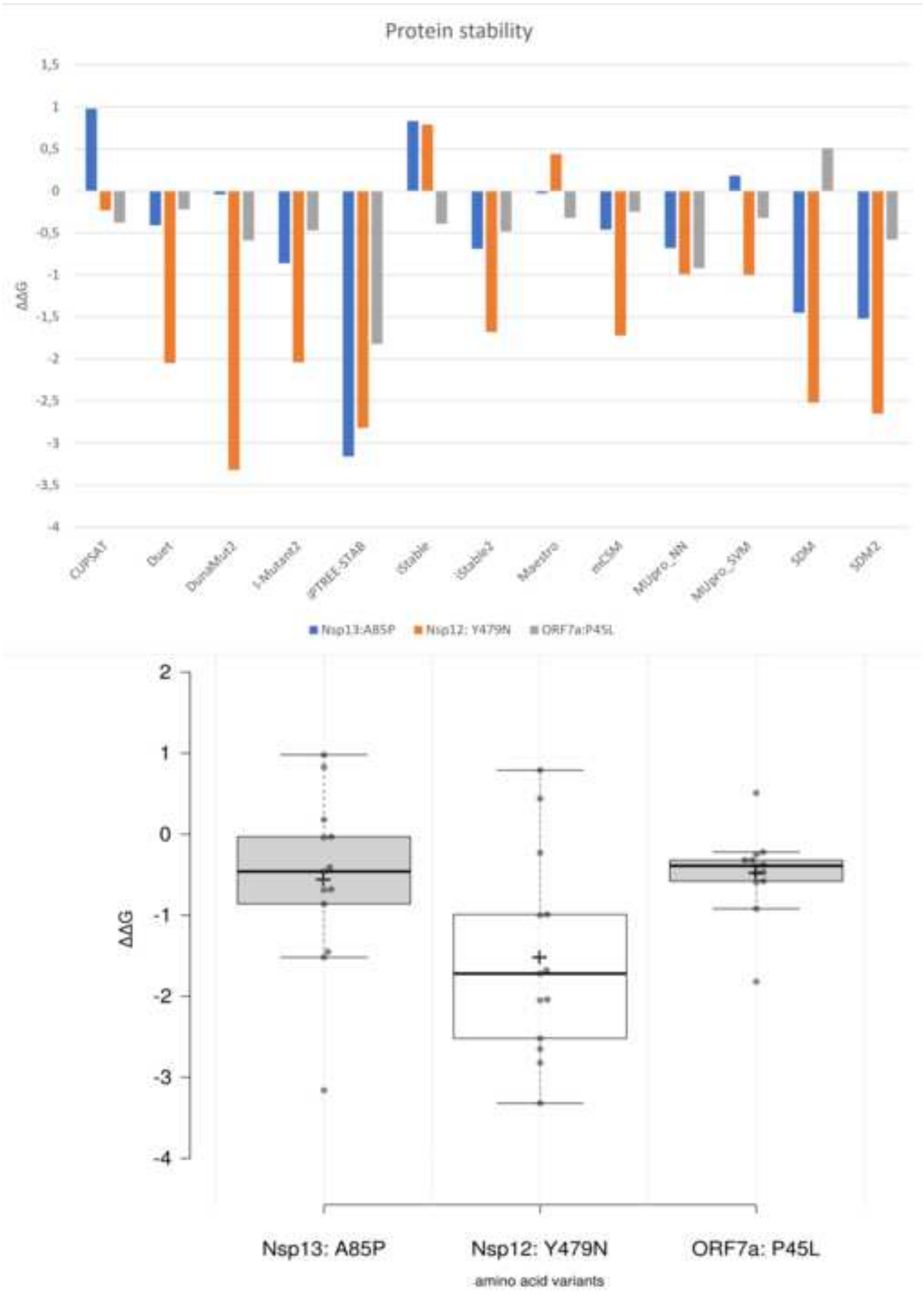

Figure 12

Click here to access/download;Figure;Fig12.tif

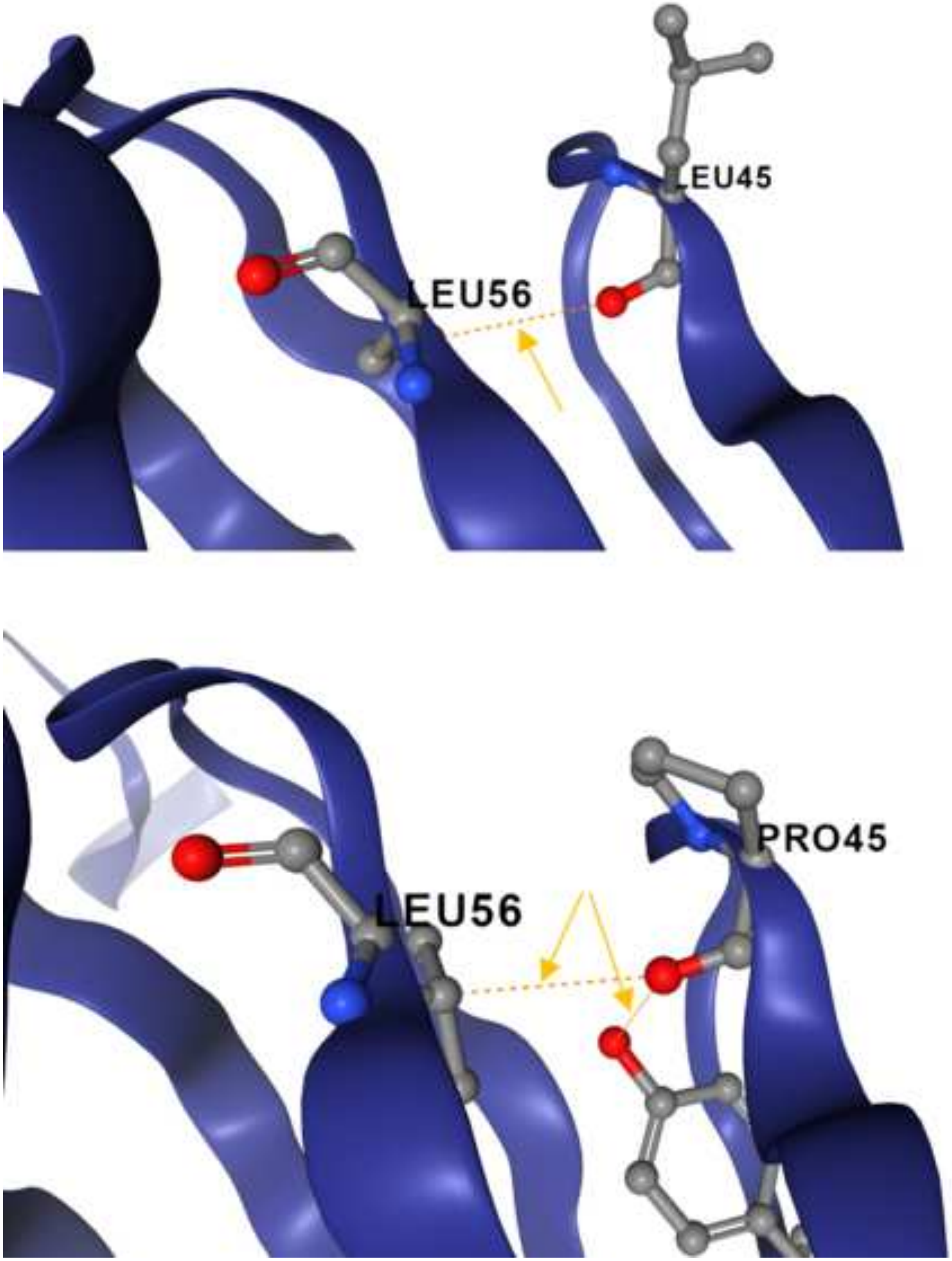

Figure 13

Click here to access/download;Figure;Fig-13-2020-2021_bootstrap.tif

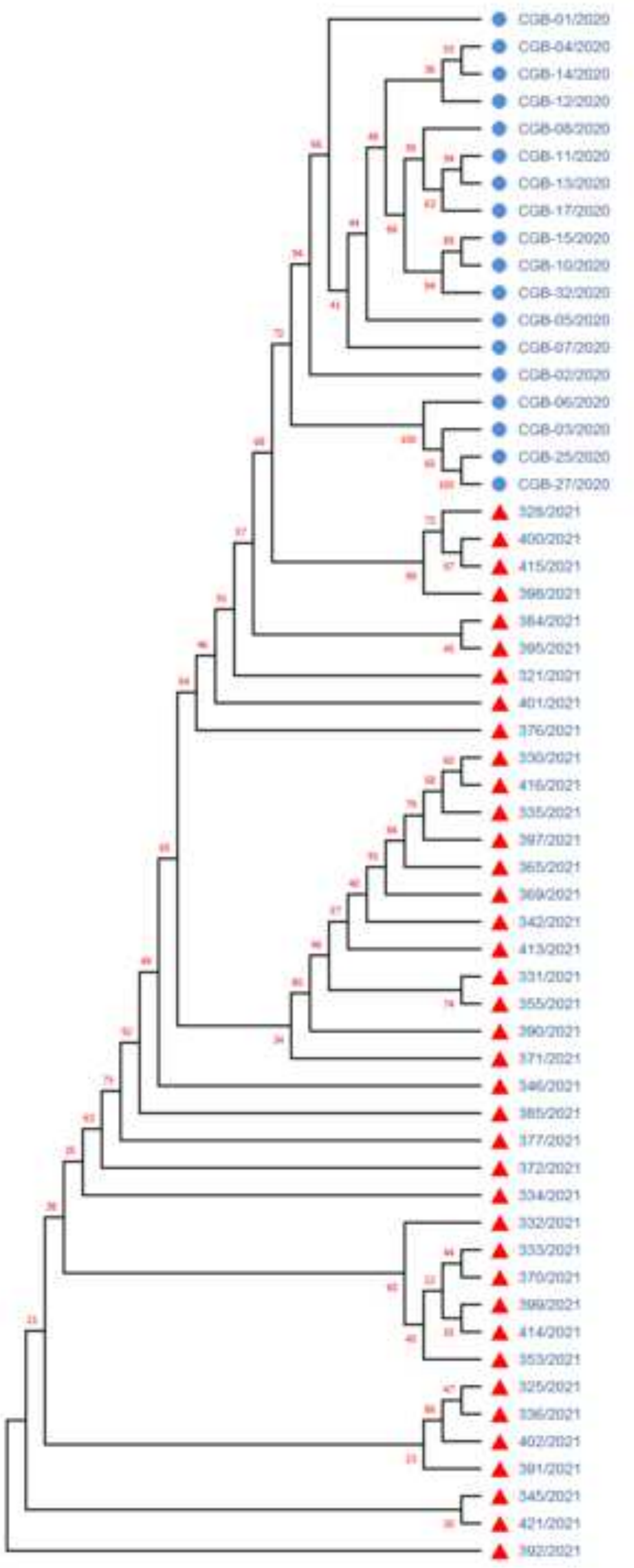

S1

Click here to access/download
Supporting Information
S1.fasta

S2

Click here to access/download
Supporting Information
S2.csv
