# Supplementary material for: Genome sequence diversity of SARS-CoV-2 obtained from clinical samples in Uzbekistan
Source: PLoS One. 2022 Jun 27;17(6):e0270314. doi: 10.1371/journal.pone.0270314 (PMC9236271; doi:10.1371/journal.pone.0270314)
Supplement: Attachment — Submitted filename: Response to reviewers.docx [file pone.0270314.s004.docx]

Response to editor and reviewers

Dear editor, thank you for valuable comments.

1. We have formatted the manuscript according to PLOS ONE's style requirements, we also have changed the order of sections, materials and methods have been moved after the introduction (see revised manuscript with track changes).

The figure files have been corrected by PACE to meet PLOS ONE requirements

2. The manuscript initially was submitted to PLOS Genetics (Manuscript ID: PGENETICS-D-22-00251) but not considered for publication by editors. Although the editors at that journal were not able to consider manuscript for publication, they encouraged us to transfer the manuscript to another PLOS journal. They have made this recommendation based on their assessment of manuscript, their knowledge of the other journal, and after consultation with other journal's editors. So, we state that the manuscript was not peer-reviewed and/or formally published elsewhere and has not been submitted simultaneously for publication elsewhere.
3. We provided repository information during submission, please hold it until acceptance. We do not wish to make changes to our Data Availability statement.
4. Thank you, we amended authorship list.
5. We will amend updated Funding Statement as follows: “This study has been supported by the research grant from the Ministry of Innovative Development, Republic of Uzbekistan (Research Grant number: А-ИРВ-2021-125). There was no additional external or internal funding received for this study. The funders had no role in study design, data collection and analysis, decision to publish, or preparation of the manuscript”. We included our amended Funding Statement within second cover letter.
6. We have updated preprints to published versions (if available) and corrected the order of references cited in the manuscript. We added one new reference and removed one (see revised manuscript with track changes)
7. According to your recommendation, a short information about possible origin of SARS-CoV-2 (citing Makarenkov et al., 2021) was added in the Introduction.
8. Thank you for suggesting a new SimPlot++ tool. We installed Windows version of this software and performed several runs. It is very useful program, but we need time to figure out which parameters suits better for analysis and how to interpret the results. For sure, we will use it to present further results, as work on collecting genomic information of SARS-CoV-2 in Uzbekistan still in progress.

Reviewer #1

Dear reviewer, we appreciate the time you spent to review our manuscript and gave valuable recommendations

1. Amino acid substitution might change protein stability, thus might increase or decrease its functional properties. In the discussion section we tried to explain possible mechanisms of amino acid substitution in ORF7a which could be a mediator of [proinflammatory cytokines](https://www.sciencedirect.com/topics/biochemistry-genetics-and-molecular-biology/proinflammatory-cytokine) production and triggers dramatic inflammatory responses resulted in disease severity.
2. N= corrected, missing value added
3. Original pictures submitted to the Journal are in good quality, after compression to pdf. the resolution is drastically reduced. I think the editor can send you the original image files by request.
4. Thank you for this suggestion, since we are continuously getting more genomic data from new samples, in our next study we will provide a detailed overview of dissimilarity of WGS of novel mutations with other mutations reported in the databases.
5. We have added references to our findings/figures in еру Discussion section as you suggested

Reviewer #2

Dear reviewer, we appreciate the time you spent to review our manuscript and gave valuable recommendations

1. We have corrected the manuscript according to your suggestions on the Pdf file
2. Conclusion phrase have been added to abstract.
3. We have made corrections in groups comparison, according to your recommendations
4. We have updated information in the Results where it was appropriate
5. The limitation has been added closer to the end of discussions as you suggested
6. The Table 1 has been transposed
7. The Table 2 has been corrected
8. The abbreviations “aa” (amino acid) and “nt” (nucleotide) have been spelled out in the text
9. The phrase “So far, during the pandemic, several factors…”. has been changed as you recommended
10. Corrected. All number smaller than has been 10 spelled out
11. We believe that Fig 13 is more suitable for discussion in its content
12. Figure 1, suggested axis names have been added
13. Figure 2, suggested axis names have been added
14. Rest figures have been corrected according to your recommendations
15. Figures with Mutation events have been changed to Violin plot style instead of Box plot
16. The image has a highest possible resolution generated by the Nexclade. The quality is better in original tif., but reduced in pdf.
17. We do not have the corona plot
